# Detecting Misspelled Drug Names Using Transformer-Based Language Models: Model Development and External Validation

**DOI:** 10.2196/91151

**Published:** 2026-09-29

**Authors:** Jiayu Lu, Kevin W McConeghy, Andrew R Zullo, Jinying Chen

**Affiliations:** 1 Department of Medicine/Section of Preventive Medicine and Epidemiology Boston University Chobanian & Avedisian School of Medicine Boston, MA United States; 2 Department of Health Services, Policy and Practice Brown University School of Public Health Providence, RI United States; 3 Transformative Health Systems Research to Improve Veteran Equity and Independence Center of Innovation (THRIVE COIN) Veterans Affairs Providence Health Care System Providence, RI United States; 4 Department of Epidemiology Brown University School of Public Health Providence, RI United States; 5 Data Science Core Boston University Chobanian & Avedisian School of Medicine Boston, MA United States

**Keywords:** misspelling detection, transformer-based language models, large language models, medication names, clinical repositories, data augmentation using RxNorm

## Abstract

**Background:**

Misspellings in medication names can compromise patient safety, reduce data utility, and impede large-scale data initiatives that integrate medication information from electronic health records (EHRs). Existing methods for detecting misspelled medical terms are mostly dictionary-based and can lead to high false-positive rates when correctly spelled but previously unseen (out-of-vocabulary) terms are encountered.

**Objective:**

We aimed to develop and validate domain-specific, transformer-based language models for detecting misspelled drug names, with an emphasis on performance for unseen terms.

**Methods:**

Using RxNorm as a standardized drug vocabulary, we created an RxNorm-augmented training corpus and developed two BERT (Bidirectional Encoder Representations from Transformers)–based models—BERTDrug and CharBERTDrug—for misspelling detection. Specifically, we randomly split 69,824 RxNorm drug names into training, development, and test sets (3:1:1) and generated k misspellings per name using text-perturbation techniques (k optimized for training; fixed at 1 for development and test sets). The models were fine-tuned on the training and development sets and evaluated using the RxNorm test set and 3586 drug names from the Long-Term Care Data Cooperative (LTCDC) database (external validation). The RxNorm test set and out-of-vocabulary LTCDC dataset (1922 terms), neither overlapping with the RxNorm training data, were used to evaluate performance on unseen terms. SpellChecker served as a dictionary-based baseline, while fastText_ML_ and BioWordVec_ML_, which used different subword embeddings as inputs for machine learning, served as additional baselines. Additionally, we compared model performance with GPT-4o, a generative large language model (LLM), using 2200 randomly sampled test terms.

**Results:**

On the RxNorm test set, BERTDrug and CharBERTDrug outperformed the baseline models across most metrics. BERTDrug achieved the best overall performance (*F*_1_-score=0.859; area under the receiver operating characteristic curve [ROC-AUC]=0.947), followed by CharBERTDrug (*F*_1_-score=0.833; ROC-AUC=0.906). Both models also outperformed the baseline models on the out-of-vocabulary LTCDC dataset across most metrics, with CharBERTDrug performing best (*F*_1_-score=0.696; ROC-AUC=0.788), followed by BERTDrug (*F*_1_-score=0.669; ROC-AUC=0.786). In the secondary analysis, both models exceeded GPT-4o on most metrics (except Recall) for 2000 RxNorm terms. BERTDrug performed best (ROC-AUC=0.951; *F*_1_-score=0.855), followed by CharBERTDrug (ROC-AUC=0.911; *F*_1_-score=0.831) and GPT-4o (ROC-AUC=0.856; *F*_1_-score=0.721). In contrast, among 200 randomly selected LTCDC terms, GPT-4o performed best on most metrics except precision and specificity.

**Conclusions:**

Domain-specific language models improved detection of misspellings in out-of-vocabulary drug names and outperformed baseline models in both internal and external evaluations. The comparison with a generative LLM suggests that domain shift may substantially reduce the advantages conferred by domain-specific training. With further fine-tuning on diverse data that capture the terminology, formatting conventions, and spelling patterns encountered across real-world clinical settings, these models could be adapted for use in other clinical databases and EHR systems to improve medication data quality for research and to support future safety-focused applications.

## Introduction

Medication names and labeling conventions are complex and susceptible to misspellings and typographical errors [1-4]. These errors can arise from several sources, including phonetic similarity and keyboard input errors (eg, adjacent key swaps, extra or missing characters, and accidental key presses). They may occur when health providers manually enter prescription orders or document treatment information in clinical notes within the electronic health record (EHR) system [5-8]. These errors can compromise patient safety and impede the effective use of EHR data for clinical research. For example, a misspelled drug name could lead to confusion about what drug should be administered and may result in a necessary treatment being held while the issue is reconciled [9]. Computerized provider order entry (CPOE) systems have been shown to significantly reduce medication prescribing errors, including typographical errors and misspellings [10,11]. However, misspelled query terms can potentially lead to search inefficiencies and CPOE-related medication errors [2,3,12]. Additionally, allowing the use of free-text instructions to enter orders within CPOE systems can further contribute to medication errors [5,12,13].

In addition to EHR systems, misspelled and nonstandard medication or drug names are commonly found in various other sources, including medical research publications [14], online surveillance systems for adverse drug events [4,15], and social media postings related to adverse drug events [16,17]. Detecting these errors is crucial for enhancing the reliability and utility of these information resources to support pharmacovigilance and medication-related research.

Furthermore, large data initiatives [18-20] aimed at advancing patient care, enabling precision medicine, and supporting comparative effectiveness research often integrate medication information from various sources, including both structured and free-text fields from EHRs. As a result, misspelled medication names may be propagated into these databases without manual review. For example, within the database of the Long-Term Care Data Cooperative (LTCDC), we found spelling errors such as *simeth****a****cone* for *simeth****i****cone*, *morphin sulfate* for *morphin****e*** *sulfate*, *lev****i****s****o****n* for *Levs****i****n*, and *Zo****rf****an* for *Zo****fr****an*. Misspelling detection is an important early step toward standardization and harmonization of such medication information.

Existing approaches to detecting misspellings in medical terms are primarily dictionary-based [2,4,6,7,15,21]. Recent research has explored the application of deep learning methods to correct misspellings in free clinical text [22,23]. However, none of them focused on medication names. Furthermore, previous studies have rarely addressed the challenge of distinguishing between misspelled medical terms and correctly spelled out-of-vocabulary terms, which can commonly arise when new medication products enter the market.

We aimed to develop and validate a deep learning approach for detecting misspelled drug names in medication orders and administration records. Our approach leveraged 2 techniques: transformer-based language models and data augmentation using RxNorm. The problem of misspelling correction can be solved in 2 steps: error detection and error correction [24]. We focused on error detection because it directly impacts the downstream task of error correction, and distinguishing between misspelled and correctly spelled out-of-vocabulary terms is itself a challenging task that warrants independent evaluation. Our results showed that RxNorm-augmented, domain-specific language models improved detection of misspellings in out-of-vocabulary drug names and outperformed both a dictionary-based baseline and 2 machine learning–based baseline models. It also outperformed GPT-4o, a generative large language model (LLM), on the internal evaluation dataset.

## Methods

### Study Design

We developed 2 transformer-based language models—BERTDrug and CharBERTDrug—to detect misspelled drug names (Figure 1). We fine-tuned the models on an augmented dataset derived from RxNorm [25] and evaluated their performance on both a held-out RxNorm-derived test set (internal validation) and drug names from the LTCDC database [20] (external validation). A dictionary-based method (SpellChecker) and 2 machine learning–based methods (fastText_ML_ and BioWordVec_ML_) served as the baselines.

**Figure 1:**
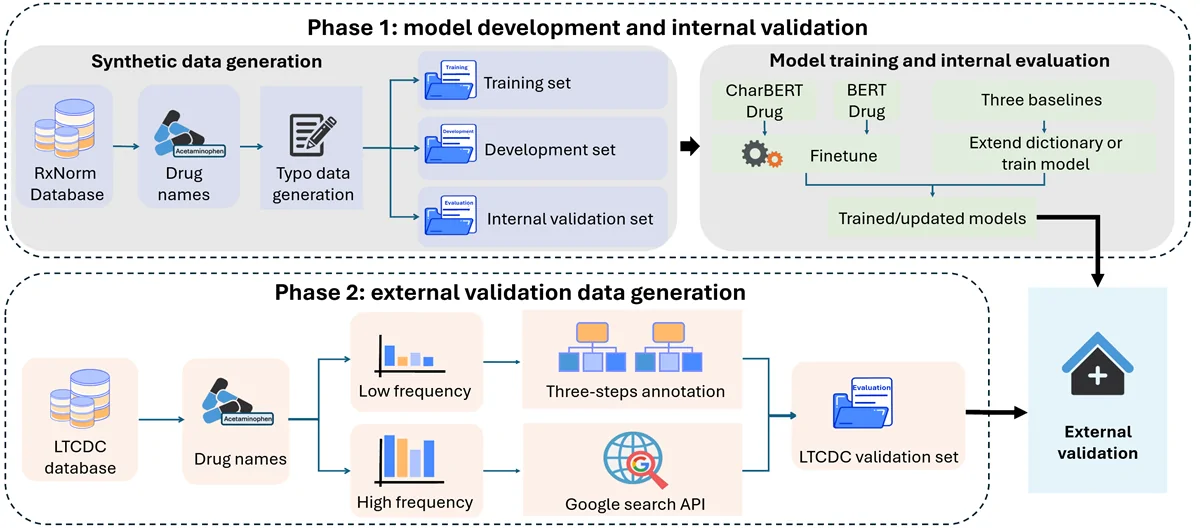
Study overview. The study was conducted in two phases: (1) development and internal validation of five models (CharBERTDrug, BERTDrug, SpellChecker, fastText_ML_, and BioWordVec_ML_) for detecting misspelled medication names, using an augmented dataset derived from RxNorm drug names, and (2) external validation using the LTCDC dataset. BERT: Bidirectional Encoder Representations from Transformers; LTCDC: Long-Term Care Data Cooperative.

### Ethical Considerations

The Institutional Review Board (IRB) of Boston University Chobanian & Avedisian School of Medicine (H-44363) reviewed the protocol for this study and determined the analysis of secondary data to be human subjects research exempt from IRB review. The IRB granted a waiver of HIPAA (Health Insurance Portability and Accountability Act) Authorization for Research under 45 CFR 164.512(i)(2)(ii). All methods were carried out in accordance with relevant guidelines and regulations.

### Data

#### Augmented Data for Model Development and Internal Validation

We used RxNorm, a large database of clinical drug information [25,26], to create a dataset for model development and internal validation. RxNorm is a normalized naming system for generic and branded clinical drugs. It standardizes drug names and maps them to drug vocabularies commonly used in pharmacy management systems. Because RxNorm is intended to represent medications and medication-related concepts rather than arbitrary nondrug terms, unrelated nondrug concepts (eg, diseases, procedures, and anatomy terms) are typically not included in RxNorm. We generated the augmented dataset in 3 steps: extracting drug names from RxNorm, preprocessing the terms (eg, converting them to lowercase), and generating misspelled drug names, as detailed in Multimedia Appendix 1. We applied text perturbation techniques—comprising the following five operations described in Zhuang and Zuccon’s study [27]—to generate synthetic positive instances with typographical errors: (1) insertion: randomly insert a letter into a word; (2) deletion: randomly delete a letter in a word; (3) substitution: randomly substitute a letter in a word; (4) swapping: randomly swap two adjacent characters in a word; and (5) adjacent keyboard character substitution: randomly replace a letter in a word with an adjacent character on the keyboard.

#### External Validation Set

We used medication names extracted from the LTCDC database [20] to externally evaluate model performance. The LTCDC database harmonized nursing home medical records from divergent EHR vendors into a common data model, including medication administrations and orders. Although most medication names were drawn from standardized databases of these EHR vendors, less common drugs could be populated by free text that was pulled into the medication name field in the LTCDC database. To ensure adequate representation of misspelled medication names in this evaluation, we used 2 complementary sampling approaches—randomly sampling from low frequency and from all LTCDC medication names, respectively. We then annotated the LTCDC datasets using a 3-phase iterative process and a semiautomatic approach. Multimedia Appendix 2 details the data sampling and annotation process.

In total, 4000 medication names from two LTCDC datasets were annotated, with 3093 negative instances (annotated as “correct drug name,” “correct nondrug name,” or “correct short drug name”), and 907 positive instances (annotated as “misspelled drug name,” “misspelled nondrug name,” or “not sure or nonstandard”). We then eliminated duplicates for 197 terms (1 positive and 196 negative) shared between LTCDC dataset-1 and dataset-2, resulting in a final LTCDC dataset of 3803 unique terms. For the primary evaluation, we derived two evaluation sets from these terms: (1) a cleaned dataset (3586 terms), which excluded nondrug terms and terms classified as “not sure” or nonstandard, and (2) a cleaned out-of-vocabulary dataset (1922 terms), which was derived from the cleaned dataset by excluding terms from the RxNorm training and development sets.

### Transformer-Based Language Models for Misspelling Detection

We fine-tuned 2 transformer-based language models, CharacterBERT_medical_ and BERT_medical_ [28], to the task of detecting misspelled drug names.

Both CharacterBERT_medical_ and BERT_medical_ models are variants of the BERT (Bidirectional Encoder Representations from Transformers) model [29]. BERT is a state-of-the-art language model for natural language processing tasks such as question answering, text classification, and machine translation [30]. It builds on the Transformer architecture and leverages the self-attention mechanism for efficient contextual understanding [31]. BERT uses a subword vocabulary for tokenization and word/token embedding. It learns the embeddings bidirectionally, from both left to right and right to left of the input sequences of tokens. BERT was designed for transfer learning, where it was first pretrained on 2 unsupervised tasks, that is, masked language modeling (MLM) and next sentence prediction (NSP), before fine-tuning for specific downstream applications such as text classification [29].

Similar to BERT, both CharacterBERT_medical_ and BERT_medical_ were pretrained on the MLM and NSP tasks, but using data from both general and biomedical domains [28]. The general-domain corpus included 5.99 million Wikipedia documents and 1.56 million OpenWebText documents, whereas the biomedical-domain corpus included 2.09 million clinical notes from the MIMIC-III (Medical Information Mart for Intensive Care III) database [32] and 2.33 million abstracts from PMC Open Access [33]. Unlike BERT, CharacterBERT_medical_ does not embed tokens (words or subwords) directly. Instead, it represents each character as a 16-dimensional real-valued vector and represents each token as a sequence of character embeddings. The character embedding sequence is passed through multiple parallel 1-dimensional convolutional neural networks (CNNs) with varying filter sizes. The outputs of these CNNs are max-pooled along the character dimension and concatenated to produce a unified representation for the sequence. The unified CNN output is then passed through 2 highway layers (which combine nonlinear transformations with residual connections) [34] and projected into a 768-dimensional space, matching the size of BERT’s word piece representation. An evaluation on medical text classification tasks, including the detection of chemical-protein interactions (ChemProt [35]) and drug-drug interactions (DDI [36]) from free text, showed that CharacterBERT_medical_ and BERT_medical_ consistently outperformed the original BERT model [28].

For this study, we developed CharBERTDrug and BERTDrug by fine-tuning CharacterBERT_medical_ and BERT_medical_ on RxNorm-augmented data (see section Model Development and Validation Using RxNorm-Augmented Data). We included BERTDrug in this study despite the primarily noncontextual nature of the misspelling detection task for two main reasons. First, many drug names are multiword expressions (eg, *abacavir-dolutegravir-lamivudine* and *Admelog SoloStar U-100 Insulin*), and BERT-based models are better able to leverage this local lexical context than noncontextual methods. Second, BERT models use subword tokenization, which, similar to character *n*-gram approaches, can help identify plausible word structure even when terms contain rare or atypical character sequences.

### Experimental Settings

#### Evaluation Metrics

We evaluated model performance by accuracy, precision, recall, specificity, *F*_1_-score, the area under the receiver operating characteristic curve (ROC-AUC), the area under the precision-recall curve (PR-AUC), and Brier score. *F*_1_-score is the harmonic mean of precision and recall. The ROC-AUC score is calculated as the area under the ROC curve, which plots true positive rate (ie, recall) against false positive rate (ie, 1-specificity). The PR-AUC score is calculated as the area under the precision-recall curve, which plots precision against recall. Compared to ROC-AUC, *F*_1_-score and PR-AUC are more sensitive to imbalanced data (eg, data with fewer positive instances than negative instances). In addition, PR-AUC provides a threshold-independent summary of performance across all classification thresholds and therefore offers a more informative assessment than *F*_1_-score when class distributions vary across settings. The Brier score measures the accuracy of predicted probabilities by calculating the mean squared difference between predicted probabilities and the actual class label (0 or 1), with lower scores indicating better performance.

#### Baseline Models

SpellChecker, a dictionary-based approach implemented using the pyspellchecker library in Python, was used as the baseline model. The SpellChecker algorithm uses dynamic programming to calculate the minimum edit distance between an input word and words in its dictionary. In this study, words present in SpellChecker’s dictionary (edit distance=0) are labeled as correctly spelled; others as misspelled. SpellChecker has shown good performance in detecting misspelled medical terms in prior studies [37,38]. To ensure a fair comparison, we extended the original dictionary of SpellChecker with the negative instances (ie, correctly spelled drug names) in the training and development splits of the RxNorm-derived dataset. Because SpellChecker does not output prediction probabilities, we did not report the Brier score for this method. As a binary classifier, SpellChecker produces only a single operating point in both the ROC and precision-recall curves. To generate multiple operating points for AUC estimation, we combined SpellChecker’s misspelling classification with term length to construct a graded decision score. Terms classified as correct by SpellChecker (ie, terms present in its dictionary) were assigned a score of 0. Among terms classified as misspelled by SpellChecker (ie, terms not present in its dictionary), longer terms were assigned higher scores, reflecting the assumption that longer terms were more likely to be misspelled. The resulting AUC scores reflect the combined measure rather than SpellChecker’s binary classification alone and should be interpreted with this limitation in mind.

In addition, we implemented 2 machine learning–based baseline models: fastText_ML_ and BioWordVec_ML_. Both models were based on the XGBoost classifier, with fastText embeddings [39] and BioWordVec embeddings [40] serving as their respective input features. FastText is a subword-based word embedding method that represents words using character *n*-grams and learns the embeddings of these subwords from a large Wikipedia-derived text corpus [39]. BioWordVec builds on the fastText framework, but its subword embeddings were trained on biomedical text derived from PubMed titles and abstracts as well as MIMIC-III clinical notes [40]. To build the fastText_ML_ model, we used the fasttext python library to learn 200-dimensional embeddings from the original RxNorm training set. In contrast, we used the pretrained BioWordVec embeddings (200-dimensional) [41] as input features for BioWordVec_ML_. For drug names containing multiple tokens, token-level embeddings were averaged element-wise using mean pooling to generate a single 200-dimensional feature vector as input to the XGBoost classifier.

#### Model Development and Validation Using RxNorm-Augmented Data

We created the training/development/test sets in two steps. First, we split the list of negative instances (ie, correctly spelled drug names from RxNorm) into a 3:1:1 ratio. Second, we created training sets of varying sizes by generating *k* positive instances (see Multimedia Appendix 1) for each negative instance in the training set and oversampling the negative instances *k* times, where *k*=1,2,4,6,8,10.

We trained CharBERTDrug and BERTDrug on each training set using 1 NVIDIA L40s GPU with an initial learning rate of 5e-5. The models were initialized using pretrained weights from CharacterBERT_medical_ and BERT_medical_, respectively. Both models were trained using the AdamW optimizer with a linear weight decay of 0.01 over 20 epochs, with a batch size of 32. To reduce the risk of overfitting, we examined the trajectory of each model’s *F*_1_-score on the development set across 0-20 epochs and selected the checkpoint with the best *F*_1_-score for subsequent evaluation (see Figure S1 in Multimedia Appendix 3). In addition, we evaluated model performance on the development set using training sets of varying sizes. For each model, the training set yielding the best performance was used in subsequent experiments. Multimedia Appendix 3 provides further information on model training and computational costs.

We trained fastText_ML_ and BioWordVec_ML_ using the original training set without oversampling. We evaluated multiple combinations of 4 XGBoost hyperparameters, including learning rate (0.03, 0.05, and 0.1), the number of trees (400 and 600), the maximum tree depth (4, 6, 8, and 10), and the subsampling rate (0.8 and 1.0), and selected the optimal configuration based on performance on the development set.

Finally, we compared the performance of CharBERTDrug, BERTDrug, and the baseline models on the test set. All machine learning–based models used a classification threshold of 0.5. In addition, we evaluated the stability of model performance under repeated resampling of the training and test sets. Specifically, we generated 20 random subsamples from the training set by repeatedly sampling 80% of the training data without replacement. Each model was retrained on the training subsamples. We then used a 2-way nonparametric bootstrap approach to estimate uncertainty in model performance and pairwise differences between classifiers (see the Additional Information on Model Evaluation section in Multimedia Appendix 3).

#### External Validation Using LTCDC Data

For the primary evaluation, we compared model performance on the cleaned LTCDC dataset and the cleaned out-of-vocabulary LTCDC dataset. For the secondary evaluation (Multimedia Appendix 4), we assessed performance on (1) the uncleaned LTCDC dataset and (2) the cleaned LTCDC dataset stratified by term type (branded vs nonbranded drug names) and term frequency (high vs low). As detailed in Multimedia Appendix 2, we sampled two LTCDC datasets (LTCDC dataset-1 and LTCDC dataset-2) from separate releases of the LTCDC database. High-frequency terms were defined as those prescribed >1000 times in dataset 1 and prescribed to >100 patients in dataset 2, while low-frequency terms were defined as those prescribed to ≤100 patients in both datasets.

To maintain a strict external evaluation setting, we applied the classifiers trained on the RxNorm data directly to the three LTCDC test sets described above, using the default classification threshold of 0.5. We used a nonparametric bootstrap approach to estimate the uncertainty in model performance and pairwise differences between classifiers on each test set (see the Additional Information on Model Evaluation section in Multimedia Appendix 4).

#### Error Analysis

Using each model’s predictions on the cleaned LTCDC terms, we identified easy and difficult cases. A term was classified as easy if its label was predicted correctly by all models, and difficult if its label was predicted incorrectly by all models.

#### Examining Factors Affecting Model Performance

CharBERTDrug and BERTDrug rely on local textual information, such as character-level or subword-level spelling patterns, to detect misspellings. Term length may affect model performance because longer terms can provide additional contextual information for the models. Term type, such as generic versus branded drug names, may also affect performance because branded names may lack consistent morphological structure and contain atypical character sequences. To quantify the impact of these factors, we used multivariable logistic regression to assess the associations of term length and term type with model performance. The outcome variable was term-level prediction accuracy, coded as 1 for a correct prediction and 0 for an incorrect prediction.

#### Comparison With an Open-Domain General-Purpose LLM

In a secondary analysis, we compared our domain-specific, transformer-based language models with a popular open-domain LLM, OpenAI’s GPT-4o (2024-08-06 snapshot), in detecting misspelled drug names. LLMs encode broad lexical and semantic knowledge acquired during pretraining, which may support recognition of drug names and detection of implausible misspellings. Although this task is primarily noncontextual, GPT-4o may still benefit from subword-level patterns and broader knowledge of biomedical terminology.

We evaluated and compared model performance on a random sample of the RxNorm-derived test set and the LTCDC dataset, which contained 1100 (RxNorm: 1000; LTCDC: 100) correctly spelled and 1100 (RxNorm: 1000; LTCDC: 100) misspelled drug names. Input was structured in batches of one drug name per prompt.

Before formal evaluation, we conducted a lightweight model selection procedure to assess the stability and performance of GPT-4o across different configurations using 250 RxNorm terms (125 negative and 125 positive instances). We evaluated four prompt styles (Table S1 in Multimedia Appendix 5): (1) baseline: user prompt only; (2) baseline + system role: the baseline prompt with an added system role; (3) enhanced instructions, which provided a more detailed task description; and (4) few-shot examples, which used example-based prompting. We also examined temperature settings of 0, 0.3, and 1.0. The model with both strong stability and good overall performance (as assessed by *F*_1_-score, ROC-AUC, and PR-AUC; detailed in Table S2 in Multimedia Appendix 5) was included in the formal evaluation.

For GPT-4o, we converted the token-level log-probabilities using NumPy’s exponential function. The positive-class probability was calculated as exp(logprob) when the generated token was “1” and as 1 − exp(logprob) when it was “0.” We then computed the ROC-AUC, PR-AUC, and Brier scores using these probability values. Because the positive-class probability was derived from the generated token’s log-probability rather than normalized over the “0” and “1” tokens, the complement of the probability for a generated “0” included residual probability assigned to other vocabulary tokens. The resulting probability estimates and metrics derived from them should therefore be interpreted cautiously.

## Results

### Descriptive Statistics of Datasets

A total of 69,824 RxNorm drug names were used for model development and internal validation, which contained 41,941 (60.1%) generic names and 27,883 (39.9%) branded names (Table 1). On average, an RxNorm drug name contains 2.4 (SD 1.5) words and 19.0 (SD 12.1) characters, with generic drug names longer than branded names (Table 1). The length of drug names is similar across the training, development, and test sets.

**Table 1:** Characteristics of RxNorm drug names (N=69,824).

			Training		Development		Test		Total
**Total number, n (%)**									
	All drug names	41,894 (60.0)		13,965 (20.0)		13,965 (20.0)		69,824 (100.0)	
	Generic names	25,218 (36.1)		8457 (12.1)		8266 (11.8)		41,941 (60.1)	
	Branded names	16,676 (23.9)		5508 (7.9)		5699 (8.2)		27,883 (39.9)	
**Length in words, mean (SD)**									
	All drug names	2.4 (1.5)		2.4 (1.5)		2.3 (1.5)		2.4 (1.5)	
	Generic names	2.5 (1.6)		2.5 (1.6)		2.5 (1.6)		2.5 (1.6)	
	Branded names	2.1 (1.4)		2.2 (1.5)		2.1 (1.4)		2.1 (1.4)	
**Length in characters, mean (SD)**									
	All drug names	19.0 (12.1)		19.0 (12.0)		18.9 (12.1)		19.0 (12.1)	
	Generic names	21.6 (12.8)		21.4 (12.6)		21.6 (12.7)		21.6 (12.8)	
	Branded names	15.0 (9.6)		15.4 (10.1)		14.8 (9.7)		15.0 (9.7)	

A total of 3803 LTCDC drug names (see Table S1 in Multimedia Appendix 2 for examples) were used for external validation of the misspelling detection models, which contained 1665 (43.8%) low-frequency names and 2138 (56.2%) high-frequency names (Table 2). Analysis of 690 misspelled LTCDC terms (see Table S2 in Multimedia Appendix 2) showed that most misspellings could be generated by our typo-creation method. Misspellings involving the exchange of 2 nonadjacent characters and phonetic-like substitutions, which could in principle be generated by our method but likely with low frequency, together accounted for approximately 10% of errors.

**Table 2:** Characteristics of LTCDC^a^ drug names (N=3803).

Category			n (%)		Number of words, mean (SD)		Number of characters, mean (SD)
**Low-frequency terms^b^ (n=1665, 43.8%)**							
	Correct drug name	620 (37.2)		1.3 (0.9)		14.0 (9.1)	
	Correct nondrug name	84 (5.0)		1.1 (0.3)		8.2 (4.0)	
	Correct short drug name	64 (3.8)		1.3 (1.0)		16.0 (9.4)	
	Misspelled drug name	799 (48.0)		1.0 (0.1)		12.7 (6.7)	
	Misspelled nondrug name	5 (0.3)		1.0 (0.0)		7.4 (0.8)	
	Not sure or nonstandard	93 (5.6)		2.1 (2.3)		16.1 (13.8)	
**High-frequency terms^c^ (n=2138, 56.2%)**							
	Correct drug name	2084 (97.5)		1.8 (1.3)		14.6 (9.4)	
	Correct nondrug name	26 (1.2)		2.3 (0.9)		14.5 (6.1)	
	Correct short drug name	19 (0.9)		2.5 (1.4)		25.2 (6.1)	
	Not sure or nonstandard	9 (0.4)		2.9 (1.3)		25.6 (11.3)	

^a^LTCDC: Long-Term Care Data Cooperative.

^b^Low frequency was defined as prescribed for ≤100 patients.

^c^High frequency was defined as prescribed >1000 times in LTCDC subset 1 and for >100 patients in LTCDC subset 2, as described in Multimedia Appendix 2.

### Internal Validation Using RxNorm-Derived Dataset

With oversampling applied to the training set and performance evaluated on the development set (see Multimedia Appendix 3: Table S1 for BERTDrug and Table S2 for CharBERTDrug), BERTDrug’s performance plateaued at an oversampling rate of 6 (*F*_1_-score=0.863; ROC-AUC=0.950; PR-AUC=0.952), while CharBERTDrug plateaued at a rate of 4 (*F*_1_-score=0.838; ROC-AUC=0.910; PR-AUC=0.907). Overall, BERTDrug outperformed CharBERTDrug across all evaluation metrics and oversampling rates.

When evaluated on the test set (Table 3), BERTDrug_6_ (model trained at an oversampling rate of 6) performed best (*F*_1_-score=0.859; ROC-AUC=0.947; PR-AUC=0.948), followed by CharBERTDrug_4_ (*F*_1_-score=0.833; ROC-AUC=0.906; PR-AUC=0.900) and fastText_ML_ (*F*_1_-score=0.795; ROC-AUC=0.846; PR-AUC=0.837). SpellChecker achieved a high recall (0.941) but the lowest precision (0.689). fastText_ML_ achieved the highest precision (0.814). Resampling-based evaluation showed that model performance remained stable and showed similar patterns of performance differences between models. The 2-way bootstrap analysis showed statistically significant differences (ie, with 95% CIs for model differences excluding 0) between each transformer model and each baseline model across most metrics. The only nonsignificant comparison was between BERTDrug and SpellChecker for recall (95% CI for difference –0.005 to 0.005). Evaluation stratified by term type showed that branded drug names were more challenging for all models (Table 3). Table S3 in Multimedia Appendix 3 provides the full results for all evaluation metrics.

**Table 3:** Model performance on RxNorm-derived test set (n=27,930), with positive instances generated using text perturbation techniques^a^.

			CharBERTDrug		BERTDrug		SpellChecker		fastText_ML_		BioWordVec_ML_
**All drug names (positive: 13,965; negative: 13,965)**											
	Precision	0.758		0.787		0.689		*0.814^g^*		0.711	
	Recall	0.925		*0.947*		0.941		0.776		0.768	
	*F*_1_-score	0.833		*0.859*		0.796		0.795		0.738	
	ROC-AUC^b^	0.906		*0.947*		0.738		0.846		0.790	
	PR-AUC^c^	0.900		*0.948*		0.652		0.837		0.757	
**Generic drug names (positive: 8266; negative: 8266)**											
	Precision	0.782		0.817		0.700		*0.831*		0.781	
	Recall	0.924		0.947		*0.949*		0.775		0.768	
	*F*_1_-score	0.847		*0.877*		0.806		0.802		0.775	
	ROC-AUC	0.921		*0.958*		0.738		0.854		0.845	
	PR-AUC	0.918		*0.961*		0.647		0.850		0.823	
**Branded drug names (positive: 5699; negative: 5699)**											
	Precision	0.730		0.751		0.676		*0.792*		0.636	
	Recall	0.927		*0.947*		0.931		0.777		0.768	
	*F*_1_-score	0.817		*0.837*		0.783		0.785		0.696	
	ROC-AUC	0.885		*0.929*		0.732		0.837		0.706	
	PR-AUC	0.874		*0.929*		0.659		0.822		0.659	
**Stability evaluation, mean (95% CI)^d,e,f^**											
	Precision	0.748 (0.742-0.754)		*0.778 (0.772-0.784)*		0.679 (0.672-0.685)		0.770 (0.762-0.777)		0.664 (0.657-0.671)	
	Recall	0.922 (0.918-0.926)		*0.942 (0.939-0.946)*		*0.942 (0.939-0.946)*		0.791 (0.783-0.798)		0.862 (0.855-0.869)	
	*F*_1_-score	0.826 (0.822-0.830)		*0.852 (0.848-0.856)*		0.789 (0.785-0.794)		0.780 (0.775-0.784)		0.750 (0.745-0.755)	
	ROC-AUC	0.898 (0.894-0.901)		*0.941 (0.938-0.943)*		0.728 (0.722-0.734)		0.834 (0.830-0.839)		0.787 (0.782-0.792)	
	PR-AUC	0.892 (0.887-0.896)		*0.942 (0.939-0.945)*		0.643 (0.635-0.651)		0.825 (0.818-0.831)		0.754 (0.748-0.761)	

^a^Positive instances were misspelled RxNorm terms generated using text perturbation techniques (eg, character insertion and deletion, swapping of adjacent characters, and substitution with keyboard-adjacent characters). The best performance score for each metric across the models was indicated in italics. For the stability evaluation, the best-performing model was determined based on the point estimates.

^b^ROC-AUC: area under the receiver operating characteristic curve.

^c^PR-AUC: area under the precision-recall curve.

^d^Model performance was evaluated using repeated resampling, with 80% of the training and test sets sampled to generate 20 random subsamples from each set.

^e^Two-way bootstrap analyses of model differences were performed for all metrics, comparing each transformer-based model with each baseline model. Most comparisons showed statistically significant differences, with 95% CIs for model differences excluding 0. Nonsignificant comparisons included BERTDrug vs SpellChecker for recall (95% CI for difference –0.005 to 0.005). Multimedia Appendix 6 provides the 95% CIs for pairwise model differences across all comparisons.

^f^Two-way bootstrap analyses compared the two transformer-based models across all metrics. All comparisons showed statistically significant differences, with 95% CIs for model differences excluding 0. Multimedia Appendix 6 provides the 95% CIs for pairwise model differences across all comparisons.

^g^The best performance score for each metric across the models is indicated in italics. For the stability evaluation, the best-performing model was determined based on the point estimates.

### External Validation Using the LTCDC Dataset

On the cleaned out-of-vocabulary LTCDC dataset (first block in Table 4 and Figure 2), CharBERTDrug achieved the best *F*_1_-score (0.696) and ROC-AUC (0.788), while BERTDrug achieved the best PR-AUC (0.665). SpellChecker achieved the highest recall (0.996). Full results for all evaluation metrics are provided in Table S1 in Multimedia Appendix 4. Bootstrap analyses of model differences showed statistically significant differences between the best-performing transformer-based model and each baseline model across most metrics, with 95% CIs of model differences excluding 0 (Table 4, note c). In contrast, differences between the two transformer-based models were not statistically significant for most metrics (Table 4, note d). Detailed model comparison results are provided in Multimedia Appendix 6. The ROC curves also demonstrated clear performance differences among the models (Figure 3). Evaluation on the cleaned full LTCDC dataset (fourth block in Table 4) and the uncleaned LTCDC dataset (Table S2 in Multimedia Appendix 4) showed similar patterns in model performance differences.

**Table 4:** Model performance on LTCDC^a^ datasets, with positive instances defined as misspelled drug names^b,c,d^.

			CharBERTDrug		BERTDrug		SpellChecker		fastText_ML_		BioWordVec_ML_
**Cleaned out-of-vocabulary dataset** **(positive: 765; negative: 1157)**											
	Precision	0.630 (0.609-0.652)		*0.631 (0.606-0.655)^g^*		0.486 (0.477-0.496)		0.381 (0.360-0.403)		0.533 (0.513-0.555)	
	Recall	0.778 (0.749-0.809)		0.712 (0.680-0.744)		*0.996 (0.991-1.000)*		0.475 (0.439-0.508)		0.708 (0.676-0.740)	
	*F*_1_-score	*0.696 (0.676-0.718)*		0.669 (0.645-0.692)		0.654 (0.645-0.663)		0.423 (0.397-0.448)		0.609 (0.587-0.630)	
	ROC-AUC^e^	*0.788 (0.767-0.808)*		0.786 (0.765-0.806)		0.646 (0.622-0.670)		0.465 (0.440-0.492)		0.655 (0.631-0.681)	
	PR-AUC^f^	0.639 (0.608-0.672)		*0.665 (0.634-0.699)*		0.457 (0.439-0.480)		0.377 (0.360-0.399)		0.544 (0.523-0.567)	
**Cleaned out-of-vocabulary dataset, generic drug names** **(positive: 461; negative: 489)**											
	Precision	*0.824 (0.794-0.855)*		0.790 (0.756-0.824)		0.603 (0.588-0.620)		0.479 (0.444-0.512)		0.649 (0.619-0.680)	
	Recall	0.783 (0.744-0.820)		0.709 (0.668-0.751)		*1.000 (1.000-1.000)*		0.464 (0.416-0.510)		0.683 (0.642-0.727)	
	*F*_1_-score	*0.803 (0.775-0.830)*		0.747 (0.715-0.779)		0.753 (0.741-0.766)		0.471 (0.432-0.506)		0.666 (0.635-0.697)	
	ROC-AUC	*0.875 (0.852-0.899)*		0.850 (0.826-0.874)		0.621 (0.583-0.659)		0.475 (0.437-0.511)		0.668 (0.634-0.703)	
	PR-AUC	0.826 (0.788-0.864)		*0.828 (0.795-0.860)*		0.533 (0.507-0.567)		0.480 (0.453-0.514)		0.651 (0.621-0.682)	
**Cleaned out-of-vocabulary dataset, branded drug names** **(positive: 304; negative: 668)**											
	Precision	0.462 (0.435-0.490)		*0.484 (0.454-0.516)*		0.375 (0.365-0.386)		0.294 (0.267-0.323)		0.427 (0.403-0.454)	
	Recall	0.770 (0.724-0.816)		0.717 (0.668-0.763)		*0.990 (0.977-1.000)*		0.490 (0.434-0.546)		0.747 (0.701-0.796)	
	*F*_1_-score	0.577 (0.548-0.607)		*0.578 (0.544-0.612)*		0.544 (0.533-0.556)		0.368 (0.333-0.403)		0.544 (0.514-0.574)	
	ROC-AUC	0.715 (0.683-0.745)		*0.733 (0.702-0.764)*		0.585 (0.550-0.618)		0.456 (0.418-0.496)		0.664 (0.631-0.698)	
	PR-AUC	0.459 (0.422-0.505)		*0.487 (0.448-0.533)*		0.334 (0.318-0.356)		0.285 (0.267-0.309)		0.447 (0.420-0.476)	
**Cleaned full dataset** **(positive: 799; negative: 2787)**											
	Precision	*0.586 (0.564-0.610)*		*0.586 (0.561-0.612)*		0.495 (0.482-0.511)		0.256 (0.240-0.272)		0.438 (0.419-0.457)	
	Recall	0.780 (0.751-0.809)		0.718 (0.686-0.748)		*0.995 (0.990-0.999)*		0.469 (0.434-0.503)		0.700 (0.668-0.731)	
	*F*_1_-score	*0.669 (0.648-0.691)*		0.645 (0.621-0.669)		0.661 (0.649-0.675)		0.331 (0.309-0.352)		0.539 (0.518-0.559)	
	ROC-AUC	0.868 (0.853-0.881)		*0.869 (0.856-0.883)*		0.850 (0.838-0.862)		0.548 (0.526-0.571)		0.748 (0.729-0.767)	
	PR-AUC	0.596 (0.564-0.632)		*0.630 (0.598-0.664)*		0.464 (0.444-0.489)		0.250 (0.236-0.266)		0.451 (0.430-0.474)	
**Cleaned full dataset, generic drug names** **(positive: 485; negative: 1197)**											
	Precision	*0.817 (0.786-0.848)*		0.783 (0.748-0.816)		0.613 (0.590-0.636)		0.341 (0.314-0.368)		0.590 (0.556-0.622)	
	Recall	0.784 (0.746-0.819)		0.713 (0.674-0.755)		*0.998 (0.994-1.000)*		0.456 (0.410-0.499)		0.672 (0.631-0.713)	
	*F*_1_-score	*0.800 (0.772-0.825)*		0.746 (0.716-0.776)		0.759 (0.742-0.777)		0.390 (0.357-0.422)		0.628 (0.596-0.660)	
	ROC-AUC	*0.918 (0.901-0.932)*		0.909 (0.893-0.925)		0.842 (0.823-0.861)		0.565 (0.535-0.594)		0.777 (0.752-0.802)	
	PR-AUC	0.806 (0.768-0.843)		*0.809 (0.776-0.843)*		0.539 (0.512-0.577)		0.338 (0.314-0.367)		0.600 (0.567-0.635)	
**Cleaned full dataset, branded drug names** **(positive: 314; negative: 1590)**											
	Precision	0.406 (0.380-0.433)		*0.424 (0.395-0.455)*		0.382 (0.365-0.400)		0.188 (0.169-0.207)		0.322 (0.300-0.344)	
	Recall	0.774 (0.726-0.822)		0.726 (0.675-0.774)		*0.990 (0.978-1.000)*		0.490 (0.433-0.545)		0.742 (0.691-0.790)	
	*F*_1_-score	0.532 (0.503-0.562)		0.535 (0.501-0.568)		*0.551 (0.534-0.570)*		0.272 (0.243-0.300)		0.449 (0.420-0.477)	
	ROC-AUC	0.825 (0.802-0.847)		*0.839 (0.816-0.859)*		0.822 (0.805-0.840)		0.534 (0.497-0.569)		0.749 (0.718-0.778)	
	PR-AUC	0.407 (0.370-0.454)		*0.445 (0.404-0.495)*		0.339 (0.320-0.366)		0.178 (0.164-0.199)		0.340 (0.313-0.369)	

^a^LTCDC: Long-Term Care Data Cooperative.

^b^Positive instances: terms labeled as “misspelled drug name”; negative instances: terms labeled as “correct drug name” and “correct short drug name.”

^c^Bootstrap analyses of model differences were performed for all metrics comparing each transformer-based model with each baseline model. Across most pairwise comparisons, the best-performing transformer-based model for each metric differed significantly from the baseline models, with 95% CIs for model differences excluding 0. Nonsignificant differences between the best-performing transformer-based model and the best-performing baseline models included BERTDrug vs BioWordVec_ML_ for PR-AUC on cleaned, branded out-of-vocabulary (OOV) terms; BERTDrug vs SpellChecker for *F*_1_-score and ROC-AUC on cleaned branded terms; and CharBERTDrug vs SpellChecker for *F*_1_-score on cleaned terms. The best performance score for each metric across the models was indicated in italics. The best-performing model was determined based on the point estimates. Multimedia Appendix 6 provides the 95% CIs for pairwise model differences across all comparisons.

^d^Bootstrap analyses compared the two transformer-based models across all metrics. Most comparisons did not show statistically significant differences, with 95% CIs for model differences including 0. Significant differences between CharBERTDrug vs BERTDrug included recall and *F*_1_-score on cleaned OOV terms; precision, recall, *F*_1_-score, and ROC-AUC on cleaned, generic OOV terms; recall on cleaned terms; and precision, recall, and *F*_1_-score on cleaned, generic terms. Multimedia Appendix 6 provides the 95% CIs for pairwise model differences across all comparisons.

^e^ROC-AUC: area under the receiver operating characteristic curve.

^f^PR-AUC: area under the precision-recall curve.

^g^The best performance score for each metric across the models was indicated in italics. The best-performing model was determined based on the point estimates.

**Figure 2:**
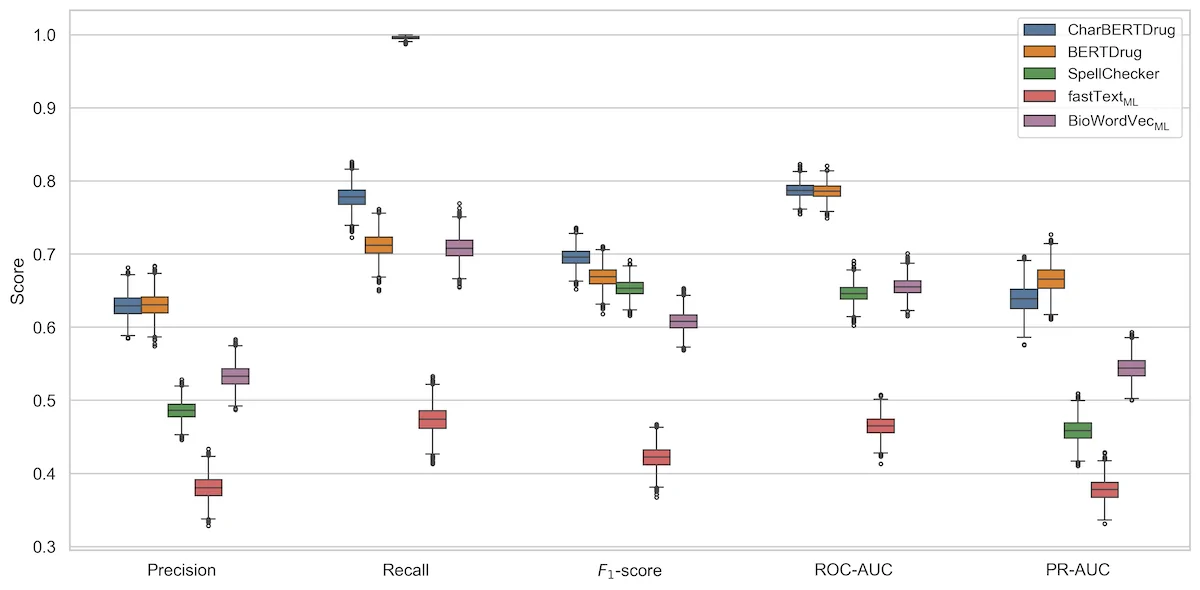
Model performance on the cleaned out-of-vocabulary LTCDC test set. LTCDC: Long-Term Care Data Cooperative; PR-AUC: area under the precision-recall curve; ROC-AUC: area under the receiver operating characteristic curve.

**Figure 3:**
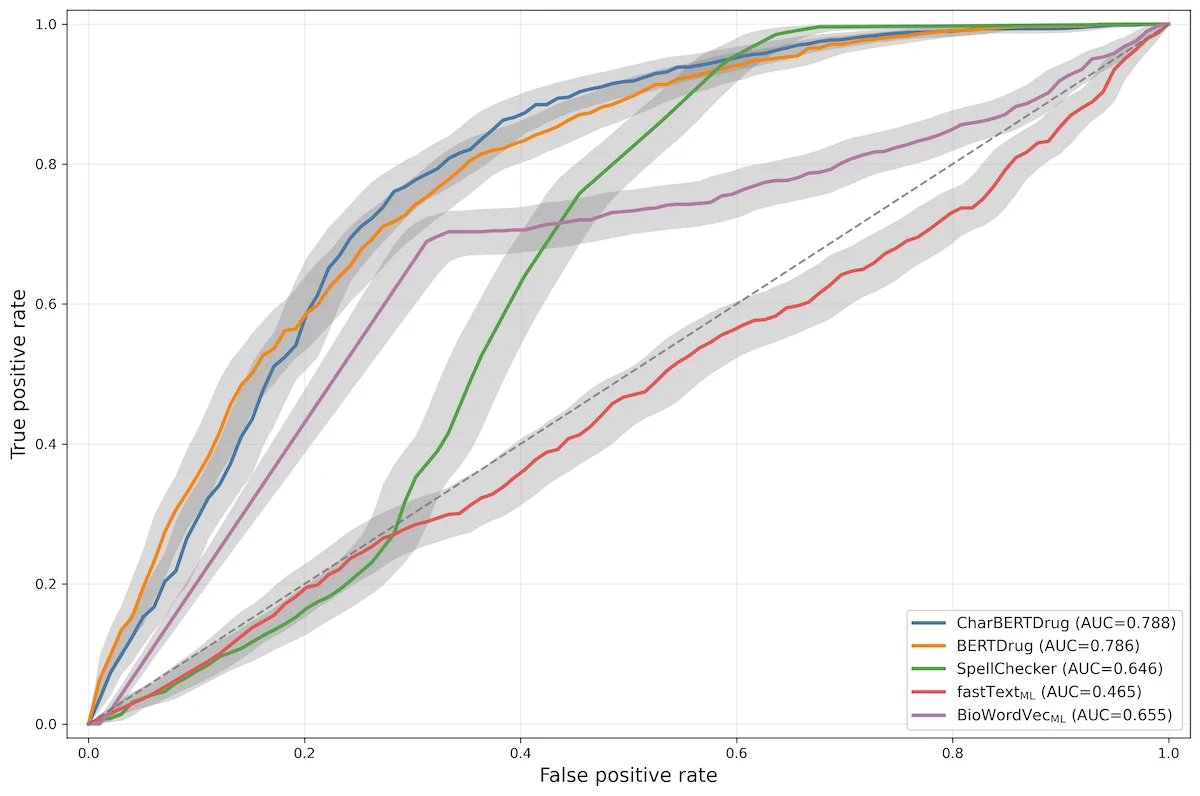
Models’ ROC curves on the cleaned out-of-vocabulary LTCDC test set. For each model, ROC curves were estimated using bootstrap resampling with 2000 replicates. In each replicate, true positive rate (TPR) values were linearly interpolated on a fixed false positive rate (FPR) grid (0-1, step=0.01). At each grid point, TPRs were aggregated across bootstrap replicates and summarized as mean and 95% CI, with the solid curve representing the mean and the shaded area indicating the 95% CI. AUC: area under the curve; LTCDC: Long-Term Care Data Cooperative; ROC: receiver operating characteristic curve.

Evaluation stratified by term type showed that branded drug names were more challenging for all models (Table 4 and Table S1 in Multimedia Appendix 4). CharBERTDrug performed best on most metrics for generic drug names in both the cleaned out-of-vocabulary set and the cleaned full set (second and fifth blocks in Table 4), whereas BERTDrug performed best on most metrics for branded drug names in both the cleaned out-of-vocabulary set and the cleaned full set (third and sixth blocks in Table 4). Bootstrap analyses of model differences showed that, for branded drug names, the two transformer-based models did not differ significantly on most metrics (Table 4, note d).

Frequency-stratified evaluation (Tables S3 and S4 in Multimedia Appendix 4) showed that CharBERTDrug performed best on high-frequency terms, while BERTDrug performed best on low-frequency terms across most metrics. The high-frequency subsets were extremely imbalanced, with no positive instances in the cleaned LTCDC dataset and only 9 (0.4% of 2138) positive instances in the uncleaned LTCDC dataset. In the uncleaned dataset, metrics sensitive to minority-class performance, including precision, *F*_1_-score, and PR-AUC, were poor across all models (eg, *F*_1_-score≤0.05), whereas accuracy and ROC-AUC were comparable to those observed for low-frequency terms (Table S4 in Multimedia Appendix 4).

### Error Analysis

A total of 3586 cleaned LTCDC terms were analyzed. Among them, 1088 (30.3%) terms were classified as easy (ie, consistently predicted correctly by all models), while 100 (2.8%) terms were difficult (ie, consistently predicted incorrectly by all models). Among the 100 difficult terms, only one term (1%), *Cyanocobalamine* instead of *Cyanocobalamin*, was a misspelled drug name that was incorrectly flagged as correct by the models. The remaining 99 (99%) difficult terms were correctly spelled but incorrectly flagged as misspellings by the models; among them, 73 (73.7%) were branded drug names, including *Hempvana* (topical product for pain relief), *Geri-kot* (senna), *Veozah* (Fezolinetant), *Zenoptiq* (ocular nutritional supplement), and more than 20 branded vaccine names (eg, *Flublok Quad 2019-2020 (PF) (flu vac qv 2019(18yr up)rc(pf))* and *Moderna COVID-19 Bival Booster)*.

Among the 128 terms that were predicted correctly by SpellChecker but incorrectly by both BERTDrug and CharBERTDrug, 72 (56.3%) were misspelled. In contrast, all 382 terms that were predicted incorrectly by SpellChecker but correctly by both BERTDrug and CharBERTDrug were correctly spelled medication names.

### Effects of Term Type and Term Length on Model Performance

As shown in Multimedia Appendix 7, in the RxNorm test set, after adjusting for term length, BERTDrug was less likely to make correct predictions for branded drug names than for generic names (odds ratio [OR] 0.883, 95% CI 0.825-0.945); while CharBERTDrug’s prediction accuracy did not differ significantly by term type (OR 0.989, 95% CI 0.928-1.054). In the LTCDC out-of-vocabulary test set, after adjusting for term length, both BERTDrug and CharBERTDrug were less likely to make correct predictions for branded drug names than for generic names (BERTDrug: OR 0.528, 95% CI 0.423-0.660; CharBERTDrug: OR 0.353, 95% CI 0.280-0.444).

Term length was positively associated with prediction accuracy in the RxNorm test set but negatively associated with prediction accuracy in the LTCDC test set (Table S1 in Multimedia Appendix 7).

### Comparison With an Open-Domain General-Purpose LLM

The GPT-4o model (baseline prompt + system role, temperature=0.0) showed strong stability and good overall performance (Table S2 in Multimedia Appendix 5) and was therefore included in the formal evaluation. Both specialized transformer-based models (ie, BERTDrug and CharBERTDrug) outperformed GPT-4o on RxNorm terms across most metrics, including *F*_1_-score (BERTDrug: 0.855, CharBERTDrug: 0.831, GPT-4o: 0.721), ROC-AUC (BERTDrug: 0.951, CharBERTDrug: 0.911, GPT-4o: 0.856), and PR-AUC (BERTDrug: 0.953, CharBERTDrug: 0.908, GPT-4o: 0.838). However, this performance advantage diminished substantially for the LTCDC terms: the transformer-based models outperformed GPT-4o only in precision and specificity, while GPT-4o performed better on all other metrics. Table S3 in Multimedia Appendix 5 provides the full results.

## Discussion

### Principal Findings

This study presents a deep-learning approach that leverages specialized, transformer-based language models to detect typographical errors in out-of-vocabulary medication names. Internal validation on the RxNorm-derived test set showed strong performance of both BERTDrug and CharBERTDrug models, with ROC-AUC scores of 0.947 and 0.906, respectively. External validation on the LTCDC terms demonstrated adequate generalizability of both models, with ROC-AUC scores of 0.786 for BERTDrug and 0.788 for CharBERTDrug on the cleaned out-of-vocabulary set and 0.869 and 0.868, respectively, on the cleaned full set.

### Related Work

Detecting misspellings from medical text has been an active area of research. Most prior studies used dictionary-based methods to detect misspelled medical terms [2,4,6,7,15,21,37]. Two recent studies have applied deep learning techniques to correct misspellings in non-English clinical texts; however, neither addressed out-of-vocabulary terms nor independently evaluated their error detection components [22,23]. To our knowledge, this study is the first to develop and evaluate deep learning models—particularly transformer-based language models—that detect misspellings from out-of-vocabulary medication names. Our approach addresses a key limitation of dictionary-based methods, which classify all out-of-vocabulary terms as misspellings and therefore suffer from excessive false positives (ie, low precision) and low specificity. For example, compared to BERTDrug, the dictionary-based SpellChecker had a significantly lower precision (0.486 vs 0.631, 95% CI for difference_BERTDrug_vs_SpellChecker_: 0.121 to 0.168) and specificity (0.304 vs 0.724, 95% CI for difference_BERTDrug_vs_SpellChecker_: 0.389 to 0.451) in detecting errors in out-of-vocabulary LTCDC medication names (refer to the highlighted rows in worksheet “Table4_Appendix4-TableS1” in Multimedia Appendix 6). This means that for every 100 correctly spelled new drug names (ie, out-of-vocabulary terms), SpellChecker generates 42 more false alarms for misspellings than BERTDrug (100 * (0.724 – 0.304)). Expanding the dictionary used by SpellChecker can improve precision and specificity, but will decrease dictionary search efficiency. More importantly, it requires ongoing monitoring of newly developed drugs and continual updates to the dictionary. In contrast, the transformer-based models (BERTDrug and CharBERTDrug) provided a superior balance across precision, specificity, and recall, along with improved overall performance as measured by *F*_1_-score and ROC-AUC. This finding highlights the advantage of using transformer-based language models for detecting misspellings in out-of-vocabulary terms. It is worth noting that even in clinical care settings—where high sensitivity in medication error detection is prioritized—a balance across precision, specificity, and recall is still crucial, particularly for hospitals that intend to deploy fully automated pipelines for error detection and correction. Excessive false alarms during error detection not only increase alert burden but may also prompt unnecessary downstream corrections, inadvertently creating new medication name errors.

Compared to the medical domain, progress in misspelling detection and correction has advanced more rapidly in the general domain. Recent research explored end-to-end approaches that leverage deep learning-based sequential models to jointly detect and correct misspellings [42-46]. Commonly used techniques included recurrent neural networks (RNNs) such as long short-term memory (LSTM) and gated recurrent unit (GRU) models [43,45], as well as transformer-based models like BERT [42,47]. Our approach is closely related to studies that used BERT-based models for error detection [44,47], but it differs in model implementation. Jayanthi and colleagues [44] applied a BERT-based model to detect and correct misspelled words. For each word in the input sequence, their model outputs a probability distribution over a predefined vocabulary. As such, their model could not handle misspellings in out-of-vocabulary words. Klemen and colleagues [47] fine-tuned the Slovene BERT model—SloBERTa [48]—for detecting misspelled words in an input sentence. They introduced a special token after each word (or after the last subword of a word) to facilitate word-level error detection. In contrast, our BERT-based models took a medication name (which was a single-word or multiple-word expression) as the input and classified it as misspelled or correctly spelled. A few prior studies have used character-based CNNs to learn local character patterns and have integrated them with RNNs to detect misspellings [44,45]. Similarly, our CharBERTDrug model combined a character-level CNN with BERT.

### Model Performance and Trade-Offs

On the cleaned full LTCDC set, CharBERTDrug achieved higher recall than BERTDrug (0.780 vs 0.718), while showing slightly lower specificity (0.842 vs 0.854). The difference likely arose from their tokenization focus: CharBERTDrug leverages character-level information, which enhances its sensitivity to subtle misspellings and boosts recall. There is a trade-off between maximizing error detection and minimizing false positives; the choice of model should be guided by the specific needs of the applications. For example, high recall in detecting misspelled medication names is preferred in applications that support pharmacovigilance and safety monitoring, whereas high precision and specificity are more desirable in real-time alerts embedded within clinical workflows, where avoiding alert fatigue is critical—particularly when late-stage quality assurance processes are available to catch additional misspellings.

Both BERTDrug and CharBERTDrug demonstrated significantly higher precision and specificity than the 3 baseline models. As shown in Figure S1 in Multimedia Appendix 8, both models had a substantially lower projected review burden per 1000 medication entries than the baseline models across plausible misspelling prevalences. As expected, SpellChecker had the lowest projected number of missed misspellings (Figure S2 in Multimedia Appendix 8), but it also incurred a substantially higher review burden than BERTDrug and CharBERTDrug (Figure S1 in Multimedia Appendix 8).

Error analysis revealed that the majority of difficult cases for all models were branded drug names that were correctly spelled but misclassified as misspellings. Association analyses also showed that the transformer-based models were less likely to make correct predictions on branded names (Multimedia Appendix 7). A possible reason is that brand names exhibit less regular spelling patterns and are therefore more difficult for the models to learn and recognize. Specifically, unlike generic drug names, brand names lack consistent morphological structure including shared prefixes or suffixes (eg, -statin, -caine, and -olol), which limits the model’s ability to generalize via subword patterns or character-level *n*-grams and forces the model to rely more heavily on memorization of individual lexical items. In addition, brand names often violate common orthographic and phonotactic patterns of natural language and contain atypical character sequences (eg, Vraylar, Xeljanz, and Qulipta) that are more likely to be interpreted by the model as noise or misspellings.

In addition to term type, term length was also associated with prediction accuracy for the two transformer-based models. However, the direction of this association differed across datasets: longer RxNorm terms were more likely to be classified correctly, whereas longer LTCDC terms were more difficult for the models to classify correctly (Multimedia Appendix 7). One possible explanation is that, although longer terms may provide additional contextual information that can benefit transformer-based models, those in the external evaluation set often contained previously unseen spelling patterns arising from combinations of formulation, strength, dosage form, and abbreviations not represented in the training data.

We also noted that, because only 9 positive instances were identified among the 2138 uncleaned high-frequency terms and all 9 positive instances were nonstandard spellings, all models performed poorly on metrics sensitive to minority-class performance (eg, precision, *F*_1_-score, and PR-AUC) on this subset. The observed pattern of low precision and moderate recall among the transformer-based models suggests that fine-tuning on nonstandard misspellings is needed to improve detection of this type of misspelling. For fully automated classification, future work could also explore hybrid strategies, such as fine-tuning separate models for high-frequency terms, when a sufficient number of positive cases are available for training, and for low-frequency terms.

Generative LLMs have demonstrated strong general-purpose capabilities, including interpreting free-text inputs and generating clinically relevant insights from vast medical knowledge [49,50]. Unlike specialized language models fine-tuned for classification tasks (eg, the models developed in this study), generative LLMs are not inherently designed as probabilistic classifiers. However, they have demonstrated strong performance on complex clinical classification tasks such as disease diagnosis [51,52]. In our secondary analysis, we found that our models outperformed the generative LLM GPT-4o in precision and speed when detecting misspelled medication names, but with lower overall classification performance than GPT-4o on LTCDC terms. These findings suggest that domain shift can substantially reduce the performance advantages of domain-specific training. Future work should improve model generalizability by incorporating more diverse training data representing the terminology, formatting conventions, and spelling patterns encountered across real-world clinical settings. Note that our analysis is preliminary, and the performance of generative LLMs could potentially be improved with more advanced models and refined prompting strategies. Future studies that systematically compare general-purpose generative LLMs and specialized language models for misspelling detection are warranted to gain deeper insights into their relative strengths and limitations.

### Limitations

This study has several limitations. First, we trained the misspelling detection models using an augmented dataset derived from RxNorm drug names, in which misspellings were introduced through commonly used text perturbation techniques. Although this approach provided a large volume of training data and improved the generalizability of the model, the generated misspellings may not fully reflect the error patterns observed in real-world databases such as LTCDC. This discrepancy likely contributed to the reduced model performance on the external validation set. Future work that incorporates misspelled medication names from real-world datasets into model training may further enhance performance. Second, the generalizability of our findings beyond the LTCDC remains uncertain. Future studies should assess performance in other contexts, such as hospital and retail pharmacy settings, where error patterns may differ. Third, our error analysis revealed that drug brand names were the most challenging cases across all models. Moreover, all models performed poorly in detecting rare misspellings (0.4%) among high-frequency drug names. Because all misspelled high-frequency terms were nonstandard spellings, this low performance is likely attributable to two factors: (1) severe class imbalance and (2) the difficulty of detecting nonstandard spellings. Specialized handling strategies or additional training data may be needed to mitigate these limitations. Fourth, the definition of high-frequency medication terms in the LTCDC external validation set was not fully consistent. LTCDC terms were sampled from two different database releases using different approaches, resulting in two separate criteria for high-frequency terms (>1000 occurrences vs >100 patients). Because earlier database releases were no longer available after LTCDC’s platform transition in late 2024, we could not repeat the original sampling process to harmonize these definitions. This inconsistency may have introduced some heterogeneity into the external evaluation. Fifth, our data augmentation strategy relied primarily on structural text perturbations (eg, insertion, deletion, and substitution). While these transformations capture many typographical errors, they do not explicitly model phonetic misspellings or improper word splitting, which are also common in real-world medication name errors. This may have limited the range of misspelling patterns represented during training and contributed to the reduced performance on the external validation set. Future work should incorporate phonetic and segmentation-based augmentation strategies, such as dropping silent-like letters, substituting similar-sounding letter groups (eg, ph versus f, c versus k), and inserting or removing spaces or hyphens in long medication names, to generate more realistic misspellings in the training data. Sixth, LTCDC terms frequently combine drug formulation, strength, dosage form, and abbreviations, making them structurally different from RxNorm drug names. This compositional shift may confound the observed association between term length and model performance in this dataset. Seventh, many medication names in the RxNorm and LTCDC datasets, or their component words, are also available through publicly accessible online resources and may have been included in the pretraining corpus of GPT-4o. Therefore, benchmarking GPT-4o on terms sampled from these datasets may introduce potential data contamination or data leakage, which could lead to an overestimation of model performance. Finally, we did not examine misspelling detection in longer clinical contexts because appropriate annotated datasets for model training and evaluation were not available. Extending this work to detect misspelled drug names within richer clinical context would be an important and practically relevant direction for future research.

### Conclusions

Our findings demonstrate that specialized language models can improve the accuracy of automated drug-name misspelling detection, particularly for out-of-vocabulary terms. When paired with downstream error correction methods, these models may further enhance medication data quality and, in future applications, support patient safety.

## Data Availability

The RxNorm database is publicly available from the U.S. National Library of Medicine. The code used to generate the synthetic dataset from RxNorm, the external validation dataset containing 3803 unique drug names from the Long-Term Care Data Cooperative database, the misspelling detection models, as well as other computer code developed for this study, are available through GitHub [53].

## Appendix

Multimedia Appendix 1 Data augmentation using RxNorm.

Multimedia Appendix 2 Creation of the LTCDC evaluation set. LTCDC: Long-Term Care Data Cooperative.

Multimedia Appendix 3 Additional information on the training and internal validation of BERTDrug and CharBERTDrug with the RxNorm-derived dataset.

Multimedia Appendix 4 Model performance on the LTCDC dataset and subsets. LTCDC: Long-Term Care Data Cooperative.

Multimedia Appendix 5 Performance comparison between CharBERTDrug, BERTDrug, and GPT-4o.

Multimedia Appendix 6 Bootstrap-based model comparisons in the RxNorm and LTCDC test sets and subsets. LTCDC: Long-Term Care Data Cooperative.

Multimedia Appendix 7 Effects of term type and term length on model performance.

Multimedia Appendix 8 Projected model outputs in real-world application scenarios.

## Abbreviations

BERT: Bidirectional Encoder Representations from Transformers
CNN: convolutional neural network
CPOE: computerized provider order entry
DDI: drug-drug interaction
EHR: electronic health record
GRU: gated recurrent unit
HIPAA: Health Insurance Portability and Accountability Act
IRB: Institutional Review Board
LLM: large language model
LSTM: long short-term memory
LTCDC: Long-Term Care Data Cooperative
MIMIC-III: Medical Information Mart for Intensive Care III
MLM: masked language modeling
NSP: next sentence prediction
OR: odds ratio
PR-AUC: area under the precision-recall curve
RNN: recurrent neural network
ROC-AUC: area under the receiver operating characteristic curve
