# Supplementary material for: Detecting Misspelled Drug Names Using Transformer-Based Language Models: Model Development and External Validation
Source: JMIR Med Inform. 2026 Sep 29;14:e91151. doi: 10.2196/91151 (PMC13662438; doi:10.2196/91151)
Supplement: Supplementary file 1 [file medinform_v14i1e91151_app1.pdf]

Multimedia Appendix 1. Data augmentation using RxNorm

We generated the RxNorm-augmented dataset in three steps.

First, we extracted drug names from the RxNorm database by filtering the TTY (Type) column (TTY="IN" or "BN") in the RXNCONSO table to select ingredient names (i.e., high-level drug names representing active substances; hereafter referred to as generic drug names) and branded names.

Second, we preprocessed these drug names by (1) converting them into lowercase; (2) removing punctuations at boundaries of the component words of each drug name; (3) splitting generic drug names that contain multiple components by "/" while handling the symbol "+/-" separately; and (4) removing duplicate drug names. Step 2 resulted in a list of 69,824 clinical drug names (41,941 generic and 27,883 brand names).

Third, we used the drug names obtained in step 2 (i.e., negative instances without typos) to generate drug names containing typos (i.e., positive instances). Specifically, we applied text perturbation techniques—comprising the following five operations described in Zhuang and Zuccon's study [1]—to generate synthetic positive instances with typos: (1) insertion: randomly insert a letter into a word; (2) deletion: randomly delete a letter in a word; (3) substitution: randomly substitute a letter in a word; (4) swapping: randomly swap two adjacent characters in a word; (5) adjacent keyboard character substitution: randomly replacing a letter in a word with an adjacent character on the keyboard.

Reference:

1. Dealing with Typos for BERT-based Passage Retrieval and Ranking; 2021 November; Online and Punta Cana, Dominican Republic. Association for Computational Linguistics.
