# Supplementary material for: Detecting Misspelled Drug Names Using Transformer-Based Language Models: Model Development and External Validation
Source: JMIR Med Inform. 2026 Sep 29;14:e91151. doi: 10.2196/91151 (PMC13662438; doi:10.2196/91151)
Supplement: Supplementary file 2 [file medinform_v14i1e91151_app2.pdf]

Multimedia Appendix 2. Creation of the LTCDC evaluation set

To ensure adequate representation of misspelled medication names in the LTCDC evaluation set, we used two complementary sampling approaches—randomly sampling from low-frequency and from all LTCDC medication names, respectively. In the first approach, we randomly extracted 2000 single-word medication names (which we called LTCDC dataset-1) from the LTCDC database (2023-12-15 release) which included 1500 low-frequency (occurred 1 or 2 times in the database) and 500 high-frequency (occurred >1000 times in the database) medication names. Note that LTCDC dataset-1 also included multi-component drug names spelled as a single word, such as *calcium-magnesium-zinc* and *cetirizine/loratadine*. In the second approach, we used a weighted sampling method to randomly select 2000 medication names (which we called LTCDC dataset-2) from a subsequent release of the LTCDC database (2024-05-23 release). The probability of each medication being sampled was proportional to its prescription frequency at the patient level, measured as the number of patients prescribed that medication. LTCDC dataset-2 contained both single-word and multi-word medication names with a wide range of patient-level prescription frequency.

We annotated the two LTCDC datasets respectively using a three-phase iterative process and a semi-automatic approach.

In the first phase, two authors (JL and JC) manually reviewed 300 low-frequency terms in LTCDC dataset-1 and assigned them into one of five categories: correct drug name, misspelled drug name, correct non-drug name, misspelled non-drug name, and not sure. The correct drug names were verified online through authentic medical websites (e.g., https://www.webmd.com/drugs/, https://www.mayoclinic.org/drugs-supplements/, etc.) or e-commerce platforms offering supplemental drugs such as multivitamins and other dietary supplements. Difficult cases were discussed with three researchers (including two coauthors KM and AZ) with expertise in pharmacy. Based on the discussion, we added another category, i.e., correct short drug name, into the final classification labels (see Table S1 for examples).

In the second phase, two authors (JL and JC) and two graduate students with training in computer science and bioinformatics annotated the 1500 low-frequency terms in LTCDC dataset-1 using the same approach used in phase 1 and assigned these terms to one of the six category labels finalized in phase 1. We assumed that the high-frequency medication names were correctly spelled and validated their correctness using the Google Custom Search API. Specifically, we wrote computer code to automatically submit the high-frequency drug names to the Google search engine through its API and check whether the search engine provided spelling suggestions. If no suggestions were present, we considered the spelling to be highly likely correct. However, to ensure annotation quality, we conducted a second-round review in which high-frequency terms were re-evaluated against the RxNorm dictionary and/or through manual verification. Specifically, terms present in RxNorm were treated as correctly spelled. For terms not found in RxNorm, we verified their correctness using authoritative medical websites or e-commerce platforms, following the same procedure described for low-frequency terms.

In the third phase, we discussed the challenging cases identified in phase 2 with the same three domain experts. Unresolved cases, e.g., those characterized by high ambiguity (e.g., *Lubrasol*, *prev*, and *Mepass*), were labeled as “not sure or non-standard”.

We annotated LTCDC dataset-2 using the same labeling scheme employed for LTCDC dataset-1. Medications prescribed to 100 or fewer patients in the LTCDC database were classified as low-frequency terms, while those prescribed to more than 100 patients were classified as high-frequency terms. The annotation process adhered to the methodology described earlier for phases 2 and 3. Note that when we applied the Google API to these terms, several were flagged as potential misspellings. We manually reviewed these flagged cases and confirmed 6 nonstandard terms. For the remaining high-frequency terms, we conducted a re-evaluation using RxNorm and manual review, following the same procedure as described for the annotation of LTCDC dataset-1 in phase 2.

After re-evaluation, among the 2,138 high-frequency terms, 2,084 (97.5%) were confirmed as correctly spelled drug names, 19 (0.9%) as correct short drug names, and 26 (1.2%) as correct non-drug names (e.g., medical devices). The remaining 9 terms (0.4%), including *Optic-Vites* and *Rileys Butt Cream*, were labeled as nonstandard.

Table S1. Example terms from the LTCDC dataset

**Category**	**Example terms**	**Note**
Correct drug name	High frequency:<br>Flexitol<br>Cozaar<br>Nizoral<br>Naratriptan HCl<br>Oral Relief Lozenges	Verified through the spell-checking function of the Google Search API, RxNorm, and manual review
	Low frequency:<br>Tranzarel<br>Dacogen<br>Ocutabs	Verified through trusted medical websites or e-commerce platforms offering supplemental drugs such as multivitamins and other dietary supplements

	FerrouSul<br>DERMACLOUD OINTMENT	
Correct short drug name	LIDO/ALUMINUM/SIMET<br><br>glucosamine/chondr/msm<br><br>EC Aspirin<br><br>Cranberry cap<br><br>Neo/poly/HC	Verified through Google search and expert review
Misspelled drug name	levison → levsin<br><br>Ketocanozole → Ketoconazole<br><br>Ducolox → Dulcolax<br><br>neomy-polymix-hc ear suspension → neomy-polymyxin-hc ear suspension<br><br>odansetron disintegrating → ondansetron disintegrating<br><br>Zyprexa sublingal → Zyprexa sublingual	Misspellings and corresponding corrections were identified using Google search and validated against trusted medical websites
Correct non-drug name [a]	CBC<br><br>glucometer<br><br>Hydroactive Dressings<br><br>Dexcom G7 Receiver<br><br>Vancouver	Google search verified that the terms are not recognized drug names.

Misspelled non-drug name	Adenovir → adenovirus<br><br>cylsis → clysis<br><br>Melaleca → Melaleuca<br><br>Tubegrip → Tubigrip	Google search verified that the terms, after spelling correction, are not recognized as drug names.
Not sure or non-standard	Wabana<br><br>Dukes<br><br>tylenol supp<br><br>aspirin 162	Any terms not in the other categories

[a] The LTCDC terms were randomly sampled from the medication_name field of the LTCDC database. This field occasionally contained common terms such as "coke," "yogurt," "mycology," and "glucometer." For these terms, it was often difficult to determine whether they represented misspelled drug names or correctly spelled non-drug terms that had been mistakenly included in the medication field during data extraction or database population. In this study, we classified them as correctly spelled non-drug terms.

Table S2. Patterns of misspelling errors in LTCDC terms (*N*=690) [a]

**Error category**	**Example terms**
**Terms with an edit distance of 1 (n=487)** [b]	
Deletion (131, 26.9%)	term: fnasteride<br>closest: finasteride
Insertion (141, 29.0%)	term: metoprolopl<br>closest: metoprolol
Substitution (185, 38.0%) [c]	

keyboard_proximity (56, 29.8%)	term: anldulafungin<br>closest: anidulafungin
phonetic_like (62, 33.0%)	term: Floraster<br>closest: Florastor
other (70, 37.2%)	term: cardesartan<br>closest: candesartan
Adjacent-character transposition (30, 6.2%)	term: erogcalciferol<br>closest: ergocalciferol
**Terms with an edit distance of 2 (n=130)** [b]	
Deletion (61, 25.5%),	term: fluocinon<br>closest: fluocinolon<br><br>term: kaylalate<br>closest: kayexalate
Insertion (61, 25.5%)	term: tizantidiine<br>closest: tizanidine<br><br>term: tricamcinelone<br>closest: triamcinolone
Substitution (89, 37.2%) [c]	
keyboard_proximity (26, 29.2%)	term: albutrtol<br>closest: albuterol

	term: arlplprazole<br>closest: aripiprazole
phonetic_like (22, 24.7%)	term: ezthromycin<br>closest: azithromycin<br><br>term: zaroxylin<br>closest: zaroxolyn
other (41, 46.1%)	term: fluctcasone<br>closest: fluticasone<br><br>term: pharylephrine<br>closest: phenylephrine
Switch (21, 8.8%)	term: collegan<br>closest: collagen<br><br>term: hydrazaline<br>closest: hydralazine
Adjacent-character transposition (7, 2.9%)	term: nitrofuantion<br>closest: nitrofurantoin<br><br>term: dounebs<br>closest: duoneb
**Terms containing only spacing or punctuation errors (n=73)** [b]	

Deletion-punctuation (8, 10.7%)	term: nuiron<br>closest: Nu-Iron
Deletion-space (58, 77.3%)	term: coughtab<br>closest: cough tab
Insertion-punctuation (9, 12.0%)	term: multiv-min-<br>closest: multi-v-min

[a] This analysis included 690 (86%) of misspelled drug names. These terms fall into three categories: (1) terms with an edit distance of 1 from the closest correctly spelled term, excluding differences involving spaces and punctuation (n=487, 70.6%); (2) terms with an edit distance of 2 from the closest correctly spelled term, again excluding spaces and punctuation (n=130, 18.8%); and (3) terms containing only spacing or punctuation errors, such as the insertion or deletion of spaces or punctuation marks (n=73, 10.6%).

[b] The total frequency of misspelling types may exceed the number of terms because individual terms may have more than one misspelling.

[c] The total frequency of the *substitution* subtypes may exceed the number of *substitution* errors because a single *substitution* error may be classified as both keyboard_proximity and phonetic_like.
