# Supplementary material for: Detecting Misspelled Drug Names Using Transformer-Based Language Models: Model Development and External Validation
Source: JMIR Med Inform. 2026 Sep 29;14:e91151. doi: 10.2196/91151 (PMC13662438; doi:10.2196/91151)
Supplement: Supplementary file 3 [file medinform_v14i1e91151_app3.pdf]

Multimedia Appendix 3. Additional information on the training and internal validation of BERTDrug and CharBERTDrug with the RxNorm-derived dataset

Table S1. BERTDrug's performance on the RxNorm development set

	**Training set size [a]**					
	**x1**	**x2**	**x4**	**x6**	**x8**	**x10**
Precision	0.899	0.854	0.825	0.791	0.792	0.773
Recall	0.795	0.880	0.924	0.950	0.950	0.963
F1	0.844	0.867	**0.872**	0.863	0.864	0.858
Accuracy	0.853	0.865	0.864	0.850	0.850	0.840
Specificity	0.911	0.849	0.804	0.750	0.750	0.718
ROC AUC	0.920	0.940	0.948	**0.950**	0.948	0.950
PR AUC	0.930	0.946	0.951	**0.952**	0.951	0.952

[a] x*k* : the training set was created by generating *k* counterparts (i.e., corresponding misspellings) for each negative instance and oversampling the negative instances *k* times, where *k*=1,2,4,6,8,10

Table S2. CharBERTDrug's performance on the RxNorm development set

	**Training set size [a]**					
**Metrics on dev set**	**x1**	**x2**	**x4**	**x6**	**x8**	**x10**
Precision	0.829	0.791	0.766	0.745	0.748	0.736
Recall	0.754	0.870	0.924	0.951	0.953	0.967
F1	0.790	0.829	**0.838**	0.835	0.838	0.836
Accuracy	0.799	0.820	0.821	0.813	0.816	0.810
Specificity	0.844	0.770	0.718	0.674	0.678	0.653
ROC AUC	0.872	0.901	**0.910**	0.910	0.907	0.901
PR AUC	0.874	0.903	**0.907**	0.902	0.879	0.859

[a] x*k* : the training set was created by generating *k* counterparts (i.e., corresponding misspellings) for each negative instance and oversampling the negative instances *k* times, where *k*=1,2,4,6,8,10

Additional Information on Model Training

To reduce risk of overfitting, we examined the trajectory of each model's F1 score on the development set across 20 epochs and selected the checkpoint with the best F1 score for subsequent evaluation (see examples in Figure S1). We chose 20 epochs as the maximum since the F1 score appeared to plateau before that point for both models.

All models were trained using a single NVIDIA L40S GPU, with peak memory usage of approximately 10 GB for both models. Training for 20 epochs required approximately 12.5 hours for BERTDrug with an oversample rate of 6 and 8 hours for CharBERTDrug with an oversample rate of 4.

Figure S1. Examples of the F1 score trajectory on the development set during model training.

a) charBERTDrug F1 score on development set, oversample rate = 4

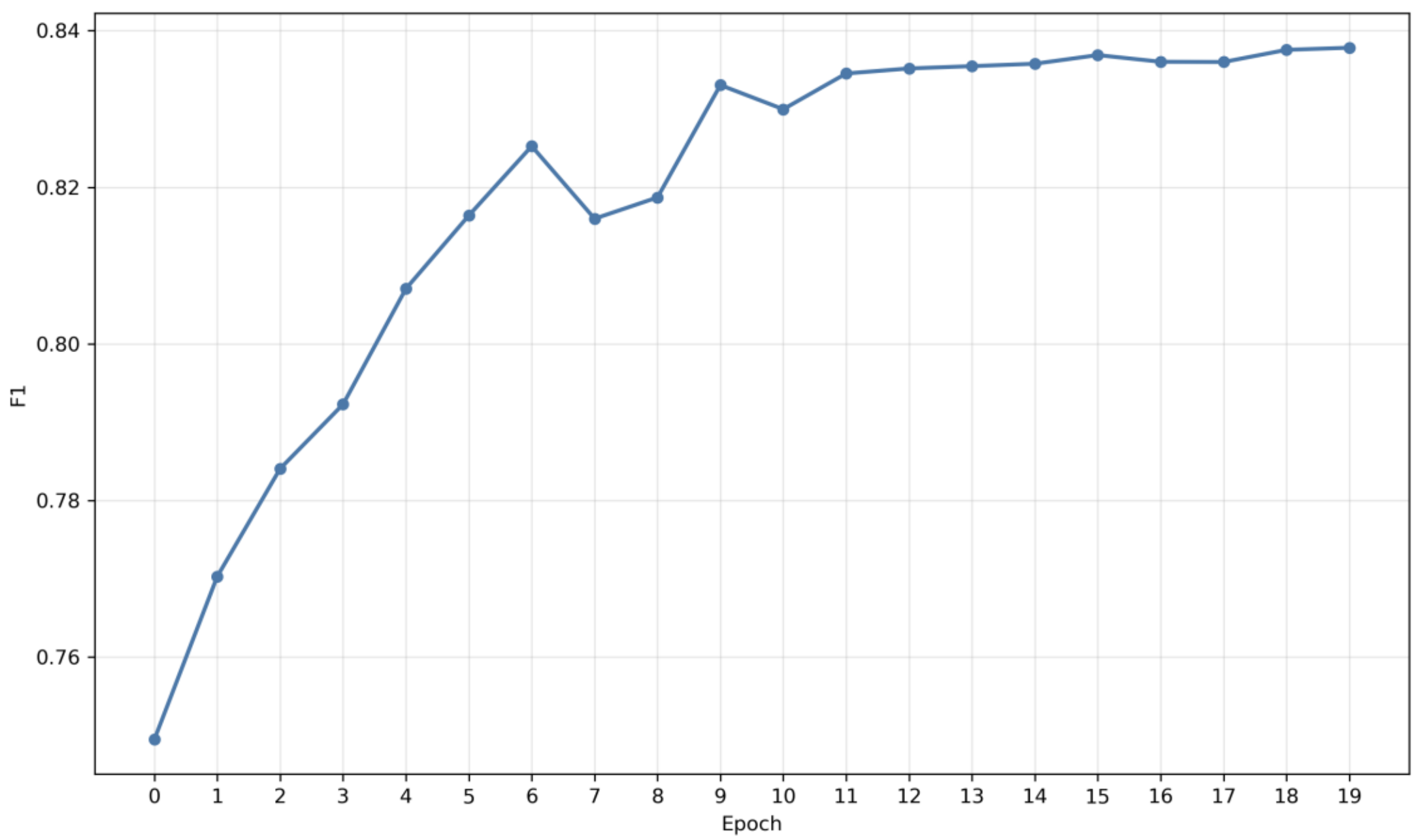

b) BERTDrug F1 score on development set, oversample rate = 6

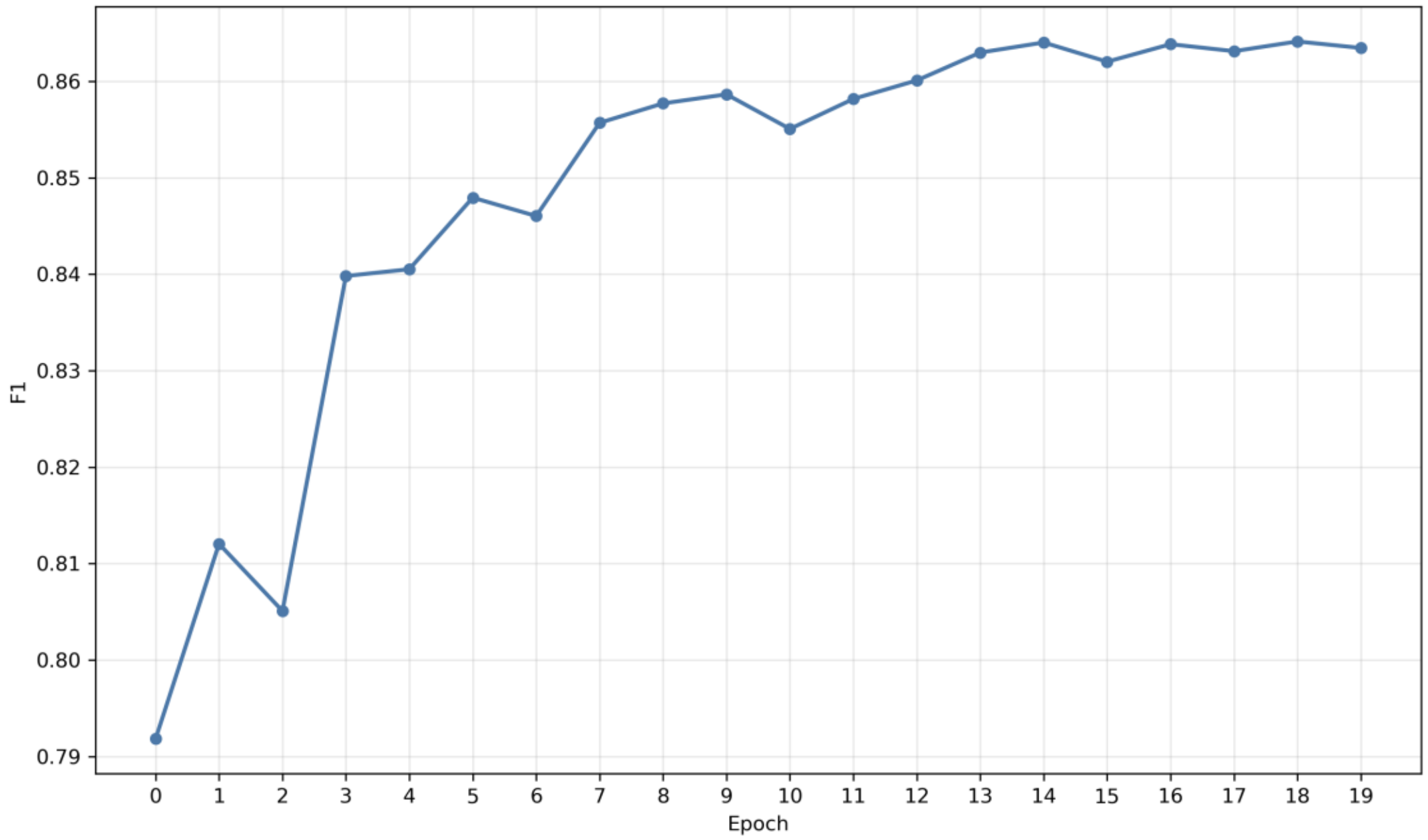

Additional Information on Model Evaluation

We used a two-way nonparametric bootstrap approach to estimate uncertainty in model performance and pairwise differences between classifiers. This approach accounts for both variation across trained models and uncertainty in the test set, without relying on independence assumptions that may be violated by overlapping resampling results.

First, 95% confidence intervals (CIs) for model performance were estimated using the following two-way bootstrap procedure. For each classifier, we trained 20 models using 20 subsamples, each consisting of 80% of the original training set. In each bootstrap iteration, we sampled model indices with replacement from the 20 trained models and sampled terms with replacement from the original test set. Each selected model was evaluated on the resampled test set, and the average of the resulting 20 performance estimates was recorded. This procedure was repeated 2,000 times to generate the bootstrap distribution for each performance metric. The empirical 2.5th and 97.5th percentiles of this distribution were used to construct the 95% CI.

Second, pairwise classifier comparisons were performed using the paired difference in performance within each bootstrap replicate. Specifically, the same resampled test set and the same resampled model indices were used for both classifiers, and the difference in their average performance was recorded for each replicate. Statistical evidence for differences between classifiers was based on the empirical bootstrap distribution of these paired performance differences, with 95% CIs derived from the empirical 2.5th and 97.5th percentiles. Differences were considered statistically significant when the 95% CI excluded zero.

Table S3. Model performance on RxNorm-derived test set (N=27,930) [a]

	CharBERTDrug	BERTDrug	SpellChecker	$fastText_{ML}$	$BioWordVec_{ML}$
**All drug names (positive: 13,965, negative: 13,965)**					
Precision	0.758	0.787	0.689	**0.814**	0.711
Recall	0.925	**0.947**	0.941	0.776	0.768
F1	0.833	**0.859**	0.796	0.795	0.738
Accuracy	0.815	**0.845**	0.759	0.799	0.728
Specificity	0.705	0.743	0.576	**0.822**	0.687
ROC-AUC	0.906	**0.947**	0.738	0.846	0.790
PR-AUC	0.900	**0.948**	0.652	0.837	0.757
Brier Score	0.169	**0.139**	–	0.182	0.184
**Generic drug names (positive: 8,266, negative: 8,266)**					
Precision	0.782	0.817	0.700	**0.831**	0.781
Recall	0.924	0.947	**0.949**	0.775	0.768
F1	0.847	**0.877**	0.806	0.802	0.775
Accuracy	0.833	**0.867**	0.771	0.809	0.777
Specificity	0.742	0.787	0.592	**0.842**	0.785
ROC-AUC	0.921	**0.958**	0.738	0.854	0.845
PR-AUC	0.918	**0.961**	0.647	0.850	0.823
Brier Score	0.151	**0.117**	–	0.173	0.160
**Branded drug names(positive: 5,699, negative: 5,699)**					
Precision	0.730	0.751	0.676	**0.792**	0.636
Recall	0.927	**0.947**	0.931	0.777	0.768

F1	0.817	0.837	0.783	0.785	0.696
Accuracy	0.792	**0.816**	0.743	0.787	0.664
Specificity	0.657	0.686	0.555	**0.796**	0.559
ROC-AUC	0.885	**0.929**	0.732	0.837	0.706
PR-AUC	0.874	**0.929**	0.659	0.822	0.659
Brier Score	0.194	**0.167**	–	0.194	0.215
**Stability evaluation, mean [95% CI] [b,c,d]**					
Precision	0.748 [0.742, 0.754]	**0.778 [0.772, 0.784]**	0.679 [0.672, 0.685]	0.770 [0.762, 0.777]	0.664 [0.657, 0.671]
Recall	0.922 [0.918, 0.926]	**0.942 [0.939, 0.946]**	**0.942 [0.939, 0.946]**	0.791 [0.783, 0.798]	0.862 [0.855, 0.869]
F1	0.826 [0.822, 0.830]	**0.852 [0.848, 0.856]**	0.789 [0.785, 0.794]	0.780 [0.775, 0.784]	0.750 [0.745, 0.755]
Accuracy	0.806 [0.801, 0.810]	**0.837 [0.833, 0.840]**	0.749 [0.744, 0.753]	0.777 [0.773, 0.781]	0.712 [0.707, 0.718]
Specificity	0.689 [0.682, 0.696]	**0.731 [0.725, 0.737]**	0.555 [0.547, 0.562]	0.763 [0.754, 0.773]	0.563 [0.553, 0.573]
ROC-AUC	0.898 [0.894, 0.901]	**0.941 [0.938, 0.943]**	0.728 [0.722, 0.734]	0.834 [0.830, 0.839]	0.787 [0.782, 0.792]
PR-AUC	0.892 [0.887, 0.896]	**0.942 [0.939, 0.945]**	0.643 [0.635, 0.651]	0.825 [0.818, 0.831]	0.754 [0.748, 0.761]
Brier Score	0.179 [0.175, 0.183]	**0.149 [0.145, 0.152]**	–	0.200 [0.196, 0.204]	0.185 [0.183, 0.187]

[a] Positive instances were misspelled RxNorm terms generated using text perturbation techniques (e.g., character insertion and deletion, swapping of adjacent characters, and substitution with keyboard-adjacent characters).

[b] Model performance was evaluated using repeated resampling, with 80% of the training and test sets sampled to generate 20 random subsamples from each set. Multimedia Appendix 6 provides the 95% CIs for pairwise model differences across all comparisons.

[c] Two-way bootstrap analyses of model differences were performed for all metrics comparing each transformer-based model with each baseline model. Most comparisons showed statistically significant differences, with 95% CIs for model differences excluding 0. Non-significant comparison(s) included: BERTDrug vs. SpellChecker for recall (95% CI for difference: -0.005 to 0.005).

[d] Two-way bootstrap analyses compared the two transformer-based models across all metrics. All comparisons showed statistically significant differences, with 95% CIs for model differences excluding 0. Multimedia Appendix 6 provides the 95% CIs for pairwise model differences across all comparisons.
