# Supplementary material for: Detecting Misspelled Drug Names Using Transformer-Based Language Models: Model Development and External Validation
Source: JMIR Med Inform. 2026 Sep 29;14:e91151. doi: 10.2196/91151 (PMC13662438; doi:10.2196/91151)
Supplement: Supplementary file 4 [file medinform_v14i1e91151_app4.pdf]

Multimedia Appendix 4. Model performance on the LTCDC dataset and subsets

Additional Information on Model Evaluation

We used a one-way nonparametric bootstrap approach to estimate uncertainty in model performance and pairwise differences between classifiers. This approach accounts for variation and uncertainty in the test set, without relying on independence assumptions that may be violated by overlapping resampling results.

First, 95% confidence intervals (CIs) for model performance were estimated as follows. In each bootstrap iteration, we sampled terms with replacement from the LTCDC test set, applied the classifiers trained on the RxNorm training set to the resampled test set, and recorded the resulting performance metrics. This procedure was repeated 2,000 times to generate the bootstrap distribution for each performance metric. The empirical 2.5th and 97.5th percentiles of this distribution were used to construct the 95% CI.

Second, pairwise classifier comparisons were performed using the paired difference in performance within each bootstrap replicate. Specifically, the same resampled test set was used for both classifiers, and the difference in performance between the two classifiers was recorded for each replicate. Statistical evidence for differences between classifiers was based on the empirical bootstrap distribution of these paired performance differences, with 95% CIs derived from the empirical 2.5th and 97.5th percentiles. Differences were considered statistically significant when the 95% CI excluded zero.

Table S1. Model performance on LTCDC datasets [a,b,c]

	**CharBERTDrug**	**BERTDrug**	**SpellChecker**	**fastText$_{ML}$**	**BioWordVec$_{ML}$**
**Cleaned out-of-vocabulary dataset (positive: 765, negative: 1157)**					
Precision	0.630 [0.609, 0.652]	**0.631 [0.606, 0.655]**	0.486 [0.477, 0.496]	0.381 [0.360, 0.403]	0.533 [0.513, 0.555]
Recall	0.778 [0.749, 0.809]	0.712 [0.680, 0.744]	**0.996 [0.991, 1.000]**	0.475 [0.439, 0.508]	0.708 [0.676, 0.740]
F1	**0.696 [0.676, 0.718]**	0.669 [0.645, 0.692]	0.654 [0.645, 0.663]	0.423 [0.397, 0.448]	0.609 [0.587, 0.630]
Accuracy	**0.729 [0.710, 0.750]**	0.720 [0.698, 0.739]	0.580 [0.563, 0.596]	0.484 [0.462, 0.506]	0.637 [0.617, 0.659]
Specificity	0.697 [0.670, 0.723]	**0.724 [0.699, 0.749]**	0.304 [0.277, 0.331]	0.490 [0.462, 0.519]	0.590 [0.562, 0.620]
ROC-AUC	**0.788 [0.767, 0.808]**	0.786 [0.765, 0.806]	0.646 [0.622, 0.670]	0.465 [0.440, 0.492]	0.655 [0.631, 0.681]
PR-AUC	0.639 [0.608, 0.672]	**0.665 [0.634, 0.699]**	0.457 [0.439, 0.480]	0.377 [0.360, 0.399]	0.544 [0.523, 0.567]
Brier Score	**0.253 [0.234, 0.271]**	0.262 [0.243, 0.281]	–	0.490 [0.469, 0.511]	0.240 [0.231, 0.250]
**Cleaned out-of-vocabulary dataset, generic drug names (positive: 461, negative: 489)**					
Precision	**0.824 [0.794, 0.855]**	0.790 [0.756, 0.824]	0.603 [0.588, 0.620]	0.479 [0.444, 0.512]	0.649 [0.619, 0.680]
Recall	0.783 [0.744, 0.820]	0.709 [0.668, 0.751]	**1.000 [1.000, 1.000]**	0.464 [0.416, 0.510]	0.683 [0.642, 0.727]
F1	**0.803 [0.775, 0.830]**	0.747 [0.715, 0.779]	0.753 [0.741, 0.766]	0.471 [0.432, 0.506]	0.666 [0.635, 0.697]
Accuracy	0.814 [0.788, 0.839]	0.767 [0.740, 0.795]	0.681 [0.660, 0.703]	0.495 [0.461, 0.526]	0.667 [0.638, 0.697]

	CharBERTDrug	BERTDrug	SpellChecker	fastText$_{ML}$	BioWordVec$_{ML}$
Specificity	**0.843 [0.810, 0.873]**	0.822 [0.787, 0.857]	0.380 [0.339, 0.423]	0.524 [0.476, 0.566]	0.652 [0.609, 0.693]
ROC-AUC	**0.875 [0.852, 0.899]**	0.850 [0.826, 0.874]	0.621 [0.583, 0.659]	0.475 [0.437, 0.511]	0.668 [0.634, 0.703]
PR-AUC	0.826 [0.788, 0.864]	**0.828 [0.795, 0.860]**	0.533 [0.507, 0.567]	0.480 [0.453, 0.514]	0.651 [0.621, 0.682]
Brier Score	**0.170 [0.147, 0.194]**	0.214 [0.189, 0.239]	–	0.479 [0.449, 0.510]	0.234 [0.220, 0.248]
**Cleaned out-of-vocabulary dataset, branded drug names (positive: 304, negative: 668)**					
Precision	0.462 [0.435, 0.490]	**0.484 [0.454, 0.516]**	0.375 [0.365, 0.386]	0.294 [0.267, 0.323]	0.427 [0.403, 0.454]
Recall	0.770 [0.724, 0.816]	0.717 [0.668, 0.763]	**0.990 [0.977, 1.000]**	0.490 [0.434, 0.546]	0.747 [0.701, 0.796]
F1	0.577 [0.548, 0.607]	**0.578 [0.544, 0.612]**	0.544 [0.533, 0.556]	0.368 [0.333, 0.403]	0.544 [0.514, 0.574]
Accuracy	0.647 [0.618, 0.677]	**0.673 [0.643, 0.702]**	0.480 [0.458, 0.503]	0.473 [0.443, 0.505]	0.608 [0.578, 0.638]
Specificity	0.591 [0.554, 0.629]	**0.653 [0.617, 0.687]**	0.249 [0.217, 0.281]	0.466 [0.430, 0.503]	0.545 [0.506, 0.582]
ROC-AUC	0.715 [0.683, 0.745]	**0.733 [0.702, 0.764]**	0.585 [0.550, 0.618]	0.456 [0.418, 0.496]	0.664 [0.631, 0.698]
PR-AUC	0.459 [0.422, 0.505]	**0.487 [0.448, 0.533]**	0.334 [0.318, 0.356]	0.285 [0.267, 0.309]	0.447 [0.420, 0.476]
Brier Score	0.334 [0.306, 0.362]	**0.309 [0.282, 0.337]**	–	0.500 [0.469, 0.530]	0.247 [0.235, 0.259]
**Cleaned full dataset (positive: 799, negative:2787)**					
Precision	**0.586 [0.564, 0.610]**	**0.586 [0.561, 0.612]**	0.495 [0.482, 0.511]	0.256 [0.240, 0.272]	0.438 [0.419, 0.457]
Recall	0.780 [0.751, 0.809]	0.718 [0.686, 0.748]	**0.995 [0.990, 0.999]**	0.469 [0.434, 0.503]	0.700 [0.668, 0.731]
F1	**0.669 [0.648, 0.691]**	0.645 [0.621, 0.669]	0.661 [0.649, 0.675]	0.331 [0.309, 0.352]	0.539 [0.518, 0.559]
Accuracy	**0.828 [0.816, 0.841]**	0.824 [0.812, 0.837]	0.773 [0.760, 0.787]	0.578 [0.562, 0.593]	0.733 [0.719, 0.747]
Specificity	0.842 [0.828, 0.856]	**0.854 [0.841, 0.868]**	0.709 [0.693, 0.727]	0.609 [0.591, 0.627]	0.742 [0.726, 0.759]
ROC-AUC	0.868 [0.853, 0.881]	**0.869 [0.856, 0.883]**	0.850 [0.838, 0.862]	0.548 [0.526, 0.571]	0.748 [0.729, 0.767]
PR-AUC	0.596 [0.564, 0.632]	**0.630 [0.598, 0.664]**	0.464 [0.444, 0.489]	0.250 [0.236, 0.266]	0.451 [0.430, 0.474]
Brier Score	**0.161 [0.149, 0.172]**	0.162 [0.150, 0.173]	–	0.397 [0.383, 0.412]	0.185 [0.179, 0.191]
**Cleaned full dataset, generic drug names (positive: 485, negative: 1197)**					
Precision	**0.817 [0.786, 0.848]**	0.783 [0.748, 0.816]	0.613 [0.590, 0.636]	0.341 [0.314, 0.368]	0.590 [0.556, 0.622]
Recall	0.784 [0.746, 0.819]	0.713 [0.674, 0.755]	**0.998 [0.994, 1.000]**	0.456 [0.410, 0.499]	0.672 [0.631, 0.713]
F1	**0.800 [0.772, 0.825]**	0.746 [0.716, 0.776]	0.759 [0.742, 0.777]	0.390 [0.357, 0.422]	0.628 [0.596, 0.660]
Accuracy	**0.887 [0.872, 0.901]**	0.860 [0.844, 0.876]	0.817 [0.800, 0.835]	0.589 [0.566, 0.612]	0.771 [0.750, 0.790]
Specificity	**0.929 [0.914, 0.943]**	0.920 [0.904, 0.934]	0.744 [0.719, 0.769]	0.643 [0.617, 0.670]	0.810 [0.788, 0.832]
ROC-AUC	**0.918 [0.901, 0.932]**	0.909 [0.893, 0.925]	0.842 [0.823, 0.861]	0.565 [0.535, 0.594]	0.777 [0.752, 0.802]
PR-AUC	0.806 [0.768, 0.843]	**0.809 [0.776, 0.843]**	0.539 [0.512, 0.577]	0.338 [0.314, 0.367]	0.600 [0.567, 0.635]
Brier Score	**0.103 [0.090, 0.117]**	0.128 [0.114, 0.143]	–	0.387 [0.366, 0.409]	0.169 [0.159, 0.178]
**Cleaned full dataset, branded drug names (positive: 314, negative: 1590)**					
Precision	0.406 [0.380, 0.433]	**0.424 [0.395, 0.455]**	0.382 [0.365, 0.400]	0.188 [0.169, 0.207]	0.322 [0.300, 0.344]
Recall	0.774 [0.726, 0.822]	0.726 [0.675, 0.774]	**0.990 [0.978, 1.000]**	0.490 [0.433, 0.545]	0.742 [0.691, 0.790]
F1	0.532 [0.503, 0.562]	0.535 [0.501, 0.568]	**0.551 [0.534, 0.570]**	0.272 [0.243, 0.300]	0.449 [0.420, 0.477]
Accuracy	0.776 [0.757, 0.795]	**0.792 [0.774, 0.810]**	0.734 [0.715, 0.753]	0.567 [0.546, 0.589]	0.700 [0.679, 0.721]
Specificity	0.776 [0.756, 0.796]	**0.805 [0.786, 0.825]**	0.683 [0.660, 0.706]	0.582 [0.558, 0.608]	0.691 [0.670, 0.714]
ROC-AUC	0.825 [0.802, 0.847]	**0.839 [0.816, 0.859]**	0.822 [0.805, 0.840]	0.534 [0.497, 0.569]	0.749 [0.718, 0.778]
PR-AUC	0.407 [0.370, 0.454]	**0.445 [0.404, 0.495]**	0.339 [0.320, 0.366]	0.178 [0.164, 0.199]	0.340 [0.313, 0.369]
Brier Score	0.212 [0.195, 0.229]	**0.192 [0.175, 0.210]**	–	0.406 [0.385, 0.427]	0.199 [0.191, 0.207]

[a] Positive instances: terms labeled as “Misspelled Drug Name”; negative instances: terms labeled as “Correct Drug Name” and “Correct Short Drug Name”.

[b] Bootstrap analyses of model differences were performed for all metrics comparing each transformer-based model with each baseline model. Across most pairwise comparisons, the best-performing transformer-based model for each metric differed significantly from the baseline models, with 95% CIs for model differences excluding 0. Non-significant differences between the best-performing transformer-based model and the best-performing baseline model(s) included: BERTDrug vs. BioWordVec$_{ML}$ for PR-AUC on cleaned, branded OOV terms; CharBERTDrug vs. BioWordVec$_{ML}$ for Brier score on cleaned OOV terms; BERTDrug vs. BioWordVec$_{ML}$ for Brier score on cleaned, branded terms; BERTDrug vs. SpellChecker for F1 and ROC-AUC on cleaned branded terms; CharBERTDrug vs. SpellChecker for F1 on cleaned terms. Multimedia Appendix 6 provides the 95% CIs for pairwise model differences across all comparisons.

[c] Bootstrap analyses compared the two transformer-based models across all metrics. Most comparisons did not show statistically significant differences, with 95% CIs for model differences including 0. Significant differences between CharBERTDrug vs. BERTDrug included: recall, F1, and specificity on cleaned OOV terms; precision, recall, F1, accuracy, ROC-AUC, and Brier score on cleaned, generic OOV terms; specificity on cleaned, branded OOV terms; recall and specificity on cleaned terms; precision, recall, F1, accuracy, and Brier score on cleaned, generic terms; specificity and Brier score on cleaned, branded terms. Multimedia Appendix 6 provides the 95% CIs for pairwise model differences across all comparisons.

Table S2. Model performance on uncleaned LTCDC dataset (All categories, *N*=3,803, positive: 906, negative: 2897) [a,b,c]

	**CharBERTDrug**	**BERTDrug**	**SpellChecker**	**fastText$_{ML}$**	**BioWordVec$_{ML}$**
Precision	0.585 [0.564, 0.608]	**0.587 [0.564, 0.610]**	0.505 [0.491, 0.519]	0.273 [0.257, 0.289]	0.442 [0.424, 0.461]
Recall	0.749 [0.722, 0.776]	0.695 [0.666, 0.725]	**0.966 [0.954, 0.977]**	0.469 [0.437, 0.502]	0.683 [0.655, 0.711]
F1	0.657 [0.636, 0.677]	0.636 [0.614, 0.659]	**0.663 [0.650, 0.676]**	0.345 [0.325, 0.366]	0.537 [0.517, 0.557]
Accuracy	**0.814 [0.801, 0.826]**	0.811 [0.798, 0.823]	0.766 [0.754, 0.778]	0.576 [0.560, 0.591]	0.719 [0.705, 0.734]
Specificity	0.834 [0.821, 0.847]	**0.847 [0.834, 0.861]**	0.704 [0.688, 0.720]	0.609 [0.591, 0.627]	0.731 [0.714, 0.747]
ROC AUC	0.844 [0.830, 0.858]	**0.854 [0.840, 0.868]**	0.833 [0.820, 0.845]	0.548 [0.527, 0.571]	0.743 [0.726, 0.761]
PR AUC	0.586 [0.557, 0.619]	**0.629 [0.599, 0.661]**	0.468 [0.449, 0.492]	0.269 [0.254, 0.288]	0.459 [0.439, 0.480]
Brier Score	0.174 [0.163, 0.185]	**0.173 [0.162, 0.184]**	–	0.400 [0.385, 0.414]	0.189 [0.183, 0.196]

[a] Positive instances: terms labeled as “Not Sure”, “Misspelled Drug Name” and “Misspelled Non-drug Name”; negative instances: terms labeled as “Correct Drug Name”, “Correct Short Drug Name”, and “Correct Non-drug Name”.

[b] Bootstrap analyses of model differences were performed for all metrics comparing each transformer-based model with each baseline model. Across most pairwise comparisons, the best-performing transformer-based model for each metric differed significantly from the baseline models, with 95% CIs for model differences excluding 0. Non-significant differences between the best-performing transformer-based model and the best-performing baseline model(s) included: CharBERTDrug vs. SpellChecker for F1. Multimedia Appendix 6 provides the 95% CIs for pairwise model differences across all comparisons.

[c] Bootstrap analyses that compared the two transformer-based models across all metrics identified significant differences for recall, specificity, and PR-AUC. Multimedia Appendix 6 provides the 95% CIs for pairwise model differences across all comparisons.

Table S3. Model performance on cleaned LTCDC dataset stratified by term frequency (N=3,586) [a,b,c]

	CharBERTDrug	BERTDrug	SpellChecker	fastText_{ML}	BioWordVec_{ML}
**Low frequency (positive: 799, negative: 684)**					
Precision	0.738 [0.716, 0.760]	**0.755 [0.732, 0.780]**	0.665 [0.651, 0.679]	0.638 [0.607, 0.671]	0.583 [0.565, 0.603]
Recall	0.780 [0.750, 0.806]	0.718 [0.688, 0.750]	**0.995 [0.989, 0.999]**	0.469 [0.436, 0.502]	0.700 [0.668, 0.731]
F1	0.758 [0.736, 0.777]	0.736 [0.714, 0.760]	**0.797 [0.787, 0.807]**	0.541 [0.510, 0.570]	0.636 [0.615, 0.658]
Accuracy	**0.732 [0.710, 0.753]**	0.723 [0.701, 0.746]	0.727 [0.710, 0.744]	0.570 [0.546, 0.595]	0.568 [0.546, 0.594]
Specificity	0.677 [0.640, 0.712]	**0.728 [0.694, 0.760]**	0.414 [0.377, 0.450]	0.689 [0.654, 0.724]	0.415 [0.379, 0.453]
ROC-AUC	0.774 [0.748, 0.797]	**0.787 [0.764, 0.810]**	0.683 [0.654, 0.712]	0.595 [0.566, 0.624]	0.563 [0.538, 0.590]
PR-AUC	0.763 [0.734, 0.793]	**0.789 [0.764, 0.815]**	0.618 [0.596, 0.646]	0.626 [0.597, 0.656]	0.584 [0.568, 0.602]
Brier Score	**0.249 [0.229, 0.270]**	0.257 [0.235, 0.278]	–	0.404 [0.380, 0.428]	0.273 [0.261, 0.284]
**High frequency (positive: 0, negative: 2103) [d]**					
Accuracy	**0.895 [0.882, 0.908]**	**0.895 [0.883, 0.909]**	0.806 [0.788, 0.822]	0.583 [0.560, 0.602]	0.849 [0.833, 0.864]
Specificity	**0.895 [0.882, 0.908]**	**0.895 [0.883, 0.909]**	0.806 [0.788, 0.822]	0.583 [0.560, 0.602]	0.849 [0.833, 0.864]
Brier score	0.099 [0.087, 0.111]	**0.095 [0.083, 0.107]**	–	0.393 [0.374, 0.414]	0.123 [0.117, 0.129]

[a] Positive instances: terms labeled as "Misspelled Drug Name"; negative instances: terms labeled as "Correct Drug Name" and "Correct Short Drug Name". Low frequency: prescribed for ≤100 patients. High frequency: prescribed >1000 times in LTCDC subset 1 and for >100 patients in LTCDC subset 2, as described in Multimedia Appendix 2.

[b] Bootstrap analyses of model differences were performed for all metrics comparing each transformer-based model with each baseline model. Across most pairwise comparisons, the best-performing transformer-based model for each metric differed significantly from the baseline models, with 95% CIs for model differences excluding 0. Non-significant differences between the best-performing transformer-based model and the best-performing baseline model(s) included: BERTDrug vs. fastText_{ML} for specificity on low-frequency terms; CharBERTDrug vs. SpellChecker for accuracy on low-frequency terms. Multimedia Appendix 6 provides the 95% CIs for pairwise model differences across all comparisons.

[c] Bootstrap analyses that compared the two transformer-based models across all metrics identified significant differences for recall and specificity on low-frequency terms. Multimedia Appendix 6 provides the 95% CIs for pairwise model differences across all comparisons.

[d] No positive instances were observed among high-frequency terms. We therefore only reported accuracy, specificity, and brier score.

Table S4. Model performance on uncleaned LTCDC datasets stratified by term frequency (N=3,803) [a,b,c]

	CharBERTDrug	BERTDrug	SpellChecker	fastText_{ML}	BioWordVec_{ML}
**Low frequency (positive: 897, negative: 768)**					
Precision	0.726 [0.704, 0.747]	**0.744 [0.721, 0.767]**	0.663 [0.649, 0.677]	0.636 [0.607, 0.666]	0.578 [0.560, 0.597]
Recall	0.751 [0.722, 0.778]	0.697 [0.667, 0.727]	**0.967 [0.954, 0.978]**	0.468 [0.437, 0.501]	0.689 [0.659, 0.718]
F1	0.738 [0.718, 0.759]	0.720 [0.697, 0.742]	**0.786 [0.775, 0.798]**	0.539 [0.510, 0.569]	0.629 [0.607, 0.649]
Accuracy	0.713 [0.691, 0.735]	0.708 [0.685, 0.729]	**0.717 [0.700, 0.735]**	0.569 [0.546, 0.594]	0.562 [0.539, 0.585]

Specificity	0.668 [0.633, 0.701]	**0.720 [0.688, 0.750]**	0.426 [0.391, 0.462]	0.688 [0.656, 0.720]	0.413 [0.378, 0.447]
ROC-AUC	0.753 [0.729, 0.776]	**0.773 [0.749, 0.795]**	0.684 [0.657, 0.711]	0.596 [0.569, 0.624]	0.567 [0.541, 0.592]
PR-AUC	0.749 [0.723, 0.778]	**0.781 [0.756, 0.805]**	0.622 [0.600, 0.649]	0.630 [0.604, 0.658]	0.587 [0.570, 0.605]
Brier Score	**0.266 [0.246, 0.287]**	0.268 [0.249, 0.289]	–	0.406 [0.383, 0.428]	0.271 [0.261, 0.282]
**High frequency (positive: 9, negative: 2129)**					
Precision	**0.022 [0.009, 0.034]**	0.021 [0.009, 0.034]	0.019 [0.014, 0.023]	0.006 [0.002, 0.009]	0.003 [0.000, 0.009]
Recall	0.556 [0.222, 0.889]	0.556 [0.222, 0.889]	**0.889 [0.667, 1.000]**	0.556 [0.222, 0.889]	0.111 [0.000, 0.333]
F1	**0.041 [0.016, 0.066]**	**0.041 [0.017, 0.066]**	0.037 [0.027, 0.044]	0.011 [0.004, 0.018]	0.006 [0.000, 0.018]
Accuracy	**0.892 [0.878, 0.906]**	0.891 [0.877, 0.904]	0.804 [0.788, 0.822]	0.580 [0.559, 0.601]	0.842 [0.826, 0.859]
Specificity	**0.893 [0.880, 0.907]**	0.892 [0.879, 0.906]	0.804 [0.787, 0.822]	0.581 [0.559, 0.601]	0.845 [0.829, 0.862]
ROC-AUC	0.638 [0.377, 0.892]	0.802 [0.588, 0.958]	**0.846 [0.719, 0.936]**	0.533 [0.343, 0.724]	0.593 [0.425, 0.756]
PR-AUC	**0.039 [0.008, 0.169]**	**0.039 [0.015, 0.101]**	0.030 [0.013, 0.099]	0.005 [0.004, 0.013]	0.006 [0.004, 0.013]
Brier score	0.102 [0.089, 0.115]	**0.098 [0.086, 0.110]**	–	0.395 [0.376, 0.414]	0.126 [0.120, 0.132]

[a] Positive instances: terms labeled as "Not Sure", "Misspelled Drug Name" and "Misspelled Non-drug Name"; negative instances: terms labeled as "Correct Drug Name", "Correct Short Drug Name", and "Correct Non-drug Name". Low frequency: prescribed for ≤100 patients. High frequency: prescribed >1000 times in LTCDC subset 1 and for >100 patients in LTCDC subset 2, as described in Multimedia Appendix 2.

[b] Bootstrap analyses of model differences were performed for all metrics comparing each transformer-based model with each baseline model. Across most pairwise comparisons, the best-performing transformer-based model differed significantly from the baseline models. Non-significant differences between the best-performing transformer-based model and the best-performing baseline model(s) included: BERTDrug vs. SpellChecker for recall, ROC-AUC, and PR-AUC on high-frequency terms; CharBERTDrug vs. SpellChecker for precision and F1 on high-frequency terms; BERTDrug vs. $\text{fastText}_{\text{ML}}$ for specificity on low-frequency terms; CharBERTDrug vs. SpellChecker for accuracy on low-frequency terms; CharBERTDrug vs. $\text{BioWordVec}_{\text{ML}}$ for Brier score on low-frequency terms. Multimedia Appendix 6 provides the 95% CIs for pairwise model differences across all comparisons.

[c] Bootstrap analyses that compared the two transformer-based models across all metrics identified significant differences for recall, specificity, and PR-AUC on low-frequency terms. Multimedia Appendix 6 provides the 95% CIs for pairwise model differences across all comparisons.
