# Supplementary material for: Detecting Misspelled Drug Names Using Transformer-Based Language Models: Model Development and External Validation
Source: JMIR Med Inform. 2026 Sep 29;14:e91151. doi: 10.2196/91151 (PMC13662438; doi:10.2196/91151)
Supplement: Supplementary file 5 [file medinform_v14i1e91151_app5.pdf]

Multimedia Appendix 5. Performance comparison between CharBERTDrug, BERTDrug, and GPT-4o

Table S1. Prompts used in different GPT-4o models

Model	Prompt
Baseline	"Classify this medication name as '0' if it is a correctly spelled medication name or '1' otherwise. Return only 0 or 1, and nothing else.\n\n{medication_name}\n"
Baseline+<br>System Role	System: "You are a medication name spelling checker. "<br>User: "Classify this medication name as '0' if it is a correctly spelled medication name or '1' otherwise. Return exactly one label and nothing else.\n\n{medication_name}\n"
Enhanced Instructions	System: You are a careful medication-name spelling auditor. Judge each line independently and do not explain your reasoning.<br>User:<br>"Task: classify this medication name with a binary label.\n"<br>"Label definitions:\n"<br>"0 = clearly a correctly spelled medication name.\n"<br>"1 = misspelled medication name, nonstandard spelling, non-medication term, noisy text, or any case where you are not confident it is a correctly spelled medication name.\n"<br>"Rules:\n"<br>"- Brand and generic medication names are valid when spelled correctly.\n"<br>"- Do not autocorrect or rewrite the input.\n"<br>"- If the name contains a typo, return 1.\n"<br>"- If uncertain, return 1.\n"<br>"- Output exactly one label: 0 or 1.\n"<br>"- Output only the label.\n\n"<br>"Medication name:\n{medication_name}\n"
Few-Shot Examples	System: "You are a careful medication-name spelling auditor. Judge spelling only and return machine-readable labels."<br><br>User:<br>"Classify this input.\n"<br>"0 = correctly spelled medication name.\n"<br>"1 = misspelled medication name, nonstandard spelling, non-medication term, noisy text, or uncertain case.\n\n"<br>"Examples:\n"<br>"metformin -> 0\n"

	"amoxicillin -> 0\n"<br>"metfornin -> 1\n"<br>"amoxcillin -> 1\n"<br>"headache -> 1\n\n"<br>"Now classify this medication name. Output exactly one label: 0 or 1. "<br>"Output only the label.\n\n"<br>"{medication_name}\n"

Table S2. Comparison of stability and performance among GPT-4o configurations [a]

Model	Consistency [b]	Precision	Recall	F1	Accuracy	Specificity	ROC-AUC	PR- AUC	Brier score
Baseline prompt, temperature = 0.0	**0.995**	0.585 ± 0.001	0.988 ± 0.004	**0.735 ± 0.002**	0.643 ± 0.002	0.298 ± 0.003	0.822 ± 0.011	0.792 ± 0.030	0.345 ± 0.002
Baseline prompt, temperature = 0.3	**0.995**	0.584 ± 0.003	0.990 ± 0.003	**0.735 ± 0.003**	0.643 ± 0.005	0.296 ± 0.010	0.822 ± 0.004	0.784 ± 0.008	0.344 ± 0.003
Baseline prompt, temperature = 1.0	0.985	0.583 ± 0.006	0.986 ± 0.003	0.733 ± 0.006	0.641 ± 0.010	0.296 ± 0.017	0.827 ± 0.007	0.800 ± 0.019	0.346 ± 0.003
Baseline+ System Role, temperature = 0.0	0.991	0.583 ± 0.003	0.992 ± 0.000	0.734 ± 0.002	0.641 ± 0.004	0.290 ± 0.009	**0.844 ± 0.004**	**0.833 ± 0.005**	0.342 ± 0.002
Baseline+ System Role, temperature = 0.3	0.991	0.584 ± 0.005	0.992 ± 0.000	**0.735 ± 0.004**	0.643 ± 0.007	0.294 ± 0.014	0.839 ± 0.003	0.819 ± 0.012	0.341 ± 0.002
Baseline+ System Role, temperature = 1.0	0.974	0.575 ± 0.007	0.984 ± 0.000	0.726 ± 0.006	0.628 ± 0.011	0.272 ± 0.022	0.842 ± 0.002	0.819 ± 0.014	0.339 ± 0.002
Enhanced Instructions, temperature = 0.0	**0.995**	0.579 ± 0.001	0.992 ± 0.000	0.731 ± 0.001	0.635 ± 0.002	0.278 ± 0.003	0.829 ± 0.004	0.782 ± 0.006	0.349 ± 0.002
Enhanced Instructions, temperature = 0.3	0.991	0.578 ± 0.003	0.988 ± 0.004	0.729 ± 0.002	0.633 ± 0.004	0.278 ± 0.010	0.829 ± 0.005	0.786 ± 0.005	0.350 ± 0.003
Enhanced Instructions, temperature = 1.0	0.975	0.575 ± 0.003	0.992 ± 0.000	0.728 ± 0.002	0.629 ± 0.004	0.266 ± 0.009	0.828 ± 0.004	0.781 ± 0.005	0.349 ± 0.003
Few shot Examples, temperature = 0.0	0.992	0.554 ± 0.004	0.992 ± 0.000	0.711 ± 0.003	0.596 ± 0.006	0.200 ± 0.013	0.770 ± 0.008	0.704 ± 0.013	0.388 ± 0.006

Few shot Examples, temperature = 0.3	0.989	0.552 ± 0.002	0.992 ± 0.000	0.709 ± 0.002	0.593 ± 0.003	0.194 ± 0.007	0.773 ± 0.008	0.708 ± 0.010	0.389 ± 0.002
Few shot Examples, temperature = 1.0	0.980	0.555 ± 0.003	0.992 ± 0.000	0.712 ± 0.002	0.598 ± 0.004	0.204 ± 0.009	0.770 ± 0.005	0.698 ± 0.008	0.391 ± 0.003

[a] Each configuration was evaluated across four repeated runs using 250 RxNorm terms, with equal numbers of positive (misspelled) and negative instances.

[b] The consistency score was defined as the average across the 250 terms of the proportion of runs that matched the majority prediction over four repetitions.

Table S3. Performance comparison between CharBERTDrug, BERTDrug, and GPT-4o [a,b]

	**CharBERTDrug**	**BERTDrug**	**GPT-4o baseline + system role, temperature = 0.0**
RxNorm terms (*N*=2000)			
Precision	0.750	**0.780**	0.565
Recall	0.930	0.945	**0.995**
F1	0.831	**0.855**	0.721
Accuracy	0.812	**0.841**	0.620
Specificity	0.698	**0.739**	0.253
ROC AUC	0.911	**0.951**	0.856
PR AUC	0.908	**0.953**	0.838
Brier Score	0.175	**0.143**	0.352
LTC terms (*N*=200)			
Precision	0.835	**0.864**	0.775
Recall	0.810	0.760	**1.000**
F1	0.822	0.809	**0.873**
Accuracy	0.825	0.820	**0.855**
Specificity	0.840	**0.880**	0.710
ROC AUC	0.868	0.906	**0.965**

PR AUC	0.816	0.883	**0.966**
Brier Score	0.168	0.160	**0.130**

[a] Model performance was evaluated using 2200 terms, which contained 1100 (RxNorm: 1000, LTCDC: 100) positive instances and 1100 (RxNorm: 1000, LTCDC: 100) negative instances. Among the LTCDC terms, positive instances were terms labeled as “Misspelled Drug Name”, whereas negative instances were terms labeled as “Correct Drug Name” and “Correct Short Drug Name”. We report model performance stratified by data source (RxNorm vs. LTCDC), noting that the results for the LTCDC terms provide comparatively noisy estimates because of the small sample size. In addition, the prevalence-sensitive metrics (precision, F1, and PR-AUC) reported for LTCDC terms are not directly comparable to the real-world results in Table 4 because of the balanced sampling.

[b] Inference Time (second/item): CharBERTDrug (<0.01 sec/term), BERTDrug (<0.01 sec/term), GPT-4o (0.2 sec/term). Both CharBERTDrug and BERTDrug were evaluated on an NVIDIA L40S GPU with Python version 3.9.21 and pytorch version 2.6.0.
