# Supplementary material for: Detecting Misspelled Drug Names Using Transformer-Based Language Models: Model Development and External Validation
Source: JMIR Med Inform. 2026 Sep 29;14:e91151. doi: 10.2196/91151 (PMC13662438; doi:10.2196/91151)
Supplement: Supplementary file 6 [file medinform_v14i1e91151_app7.pdf]

Multimedia Appendix 7. Effects of term type and term length on model performance

Table S1. Associations of term type and term length with term-level prediction accuracy [a]

	BERTDrug		CharBERTDrug	
	Odds Ratio (95% CI)	*P* value	Odds Ratio (95% CI)	*P* value
**RxNorm test set**				
Term type (branded vs. generic names)	0.883 (0.825, 0.945)	<.001*	0.989 (0.928, 1.054)	.7
Term length (in characters)	1.045 (1.041, 1.049)	<.001*	1.044 (1.040, 1.047)	<.001*
**LTCDC out-of-vocabulary test set**				
Term type (branded vs. generic names)	0.528 (0.423, 0.660)	<.001*	0.353 (0.280, 0.444)	<.001*
Term length (in characters)	0.980 (0.971, 0.990)	<.001*	0.977 (0.966, 0.987)	<.001*

[a] Associations were assessed using multivariable logistic regression, with term type and term length as predictor variables and term-level prediction accuracy as the outcome. Term type was coded as a binary variable (1: brand-name, 0: generic drug name), with generic drug name as the reference category. Term length was measured in characters. The outcome variable was coded as 1 for a correct prediction and 0 for an incorrect prediction.
* indicates statistically significant at $P < .05$.
