# Supplementary material for: Detecting Misspelled Drug Names Using Transformer-Based Language Models: Model Development and External Validation
Source: JMIR Med Inform. 2026 Sep 29;14:e91151. doi: 10.2196/91151 (PMC13662438; doi:10.2196/91151)

Multimedia Appendix 8. Projected model outputs in real-world application scenarios

Figure S1. Projected review burden (true positives plus false positives) per 1,000 medication entries across plausible misspelling prevalence levels. Projections were calculated using each model's recall and specificity on the cleaned LTCDC dataset.

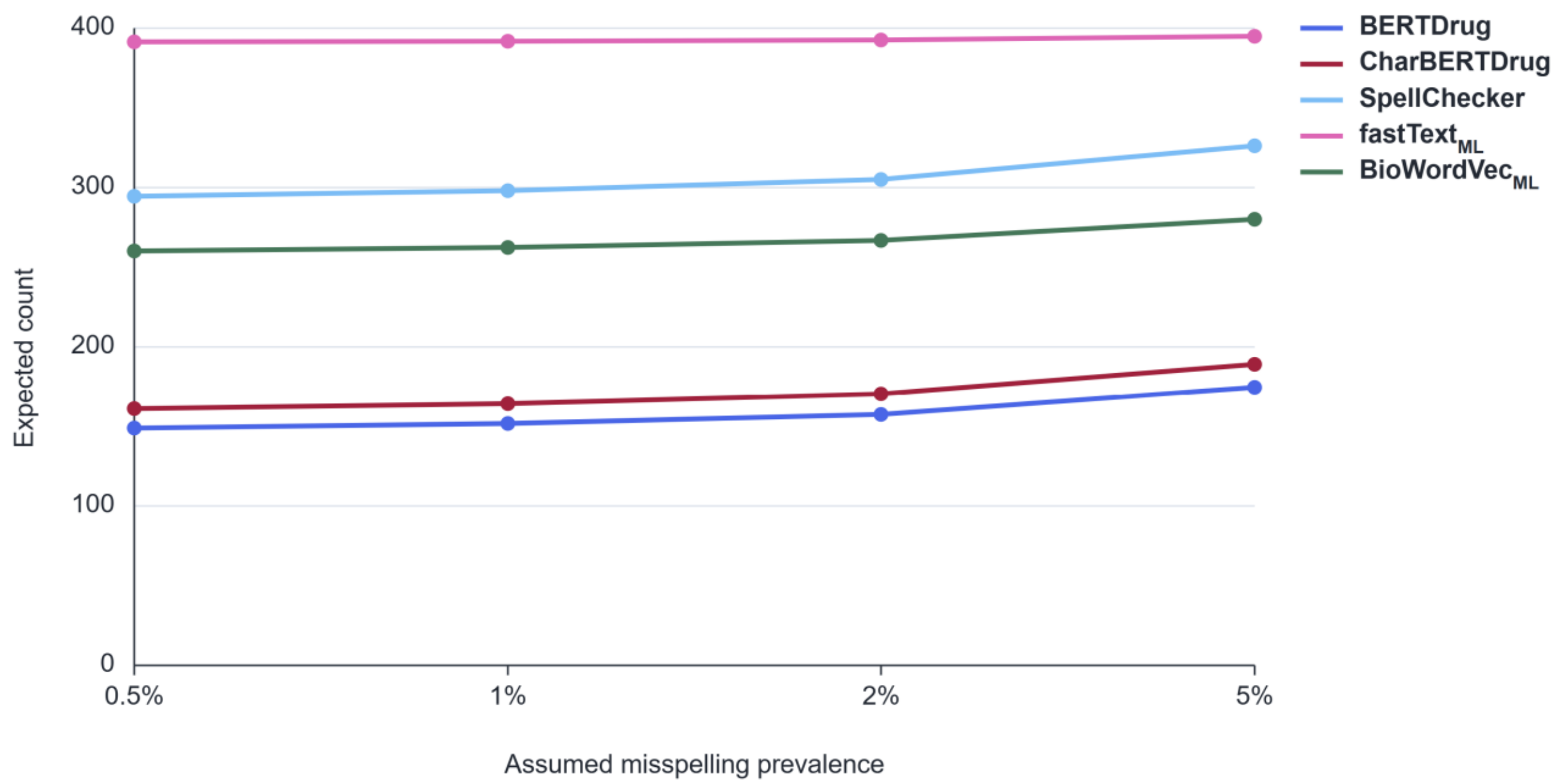

Figure S2. Projected number of missed misspellings (false negatives) per 1,000 medication entries across plausible misspelling prevalence levels. Projections were calculated using each model's recall on the cleaned LTCDC dataset.

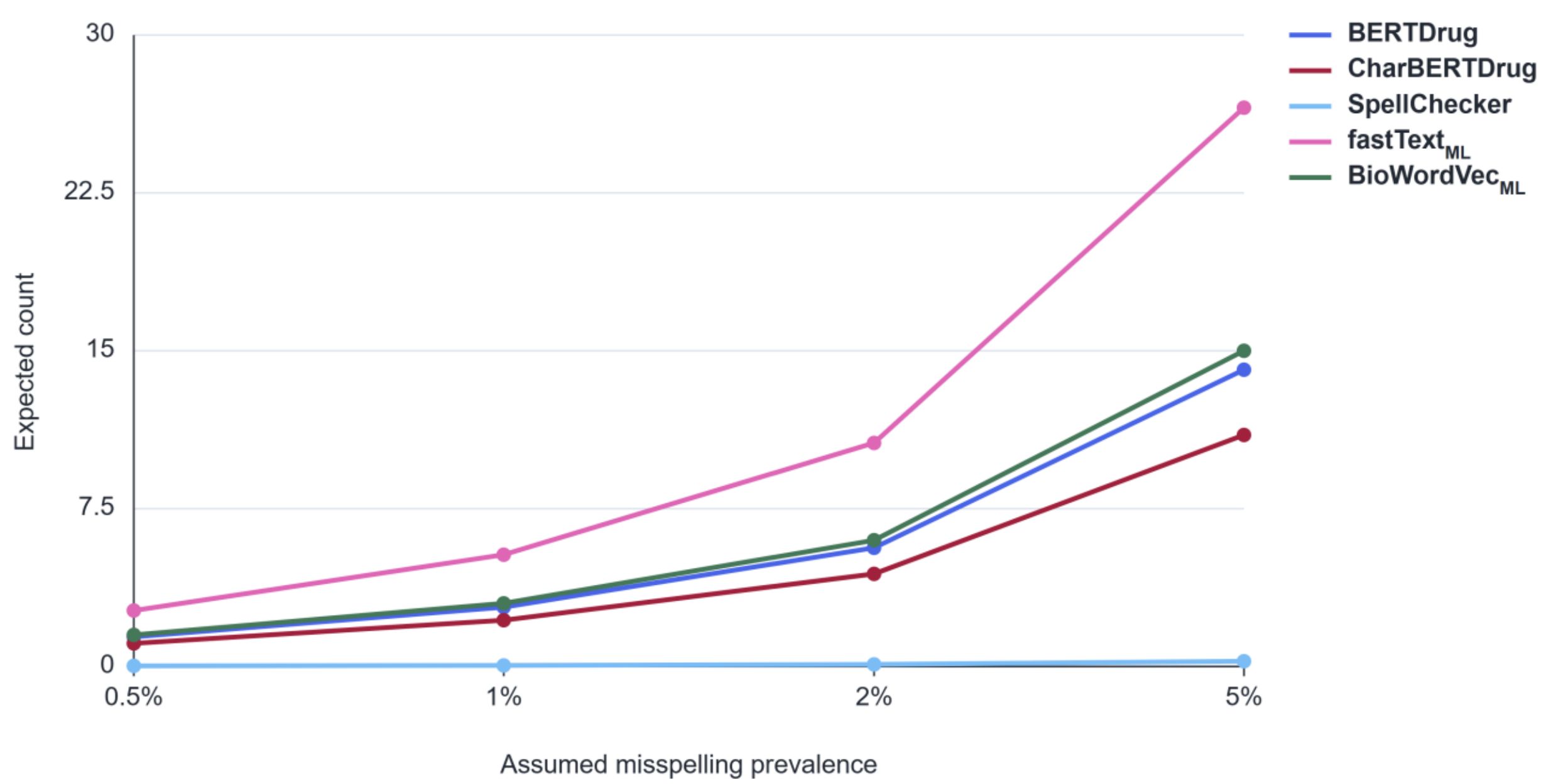

Supplement: Supplementary file 7 [file medinform_v14i1e91151_app8.pdf]
